# *AMFR* dysfunction causes autosomal recessive spastic paraplegia in human that is amenable to statin treatment in a preclinical model

**DOI:** 10.1007/s00401-023-02579-9

**Published:** 2023-04-29

**Authors:** Ruizhi Deng, Eva Medico-Salsench, Anita Nikoncuk, Reshmi Ramakrishnan, Kristina Lanko, Nikolas A. Kühn, Herma C. van der Linde, Sarah Lor-Zade, Fatimah Albuainain, Yuwei Shi, Soheil Yousefi, Ivan Capo, Evita Medici van den Herik, Marjon van Slegtenhorst, Rick van Minkelen, Geert Geeven, Monique T. Mulder, George J. G. Ruijter, Dieter Lütjohann, Edwin H. Jacobs, Henry Houlden, Alistair T. Pagnamenta, Kay Metcalfe, Adam Jackson, Siddharth Banka, Lenika De Simone, Abigail Schwaede, Nancy Kuntz, Timothy Blake Palculict, Safdar Abbas, Muhammad Umair, Mohammed AlMuhaizea, Dilek Colak, Hanan AlQudairy, Maysoon Alsagob, Catarina Pereira, Roberta Trunzo, Vasiliki Karageorgou, Aida M. Bertoli-Avella, Peter Bauer, Arjan Bouman, Lies H. Hoefsloot, Tjakko J. van Ham, Mahmoud Issa, Maha S. Zaki, Joseph G. Gleeson, Rob Willemsen, Namik Kaya, Stefan T. Arold, Reza Maroofian, Leslie E. Sanderson, Tahsin Stefan Barakat

**Affiliations:** 1 Department of Clinical Genetics, Erasmus MC University Medical Center, Rotterdam, The Netherlands; 2 Whole Genome Sequencing Implementation and Research Task Force, Department of Clinical Genetics, Erasmus MC University Medical Center, Rotterdam, The Netherlands; 3 Bioscience Program, Biological and Environmental Science and Engineering Division, Computational Bioscience Research Center, King Abdullah University of Science and Technology (KAUST), Thuwal, 23955-6900 Saudi Arabia; 4 Department of Cell Biology, Erasmus MC University Medical Center, Rotterdam, The Netherlands; 5 Department for Histology and Embryology, Faculty of Medicine, University of Novi Sad, Novi Sad, Serbia; 6 Department of Neurology, Erasmus MC University Medical Center, Rotterdam, The Netherlands; 7 Department of Internal Medicine, Erasmus MC University Medical Center, Rotterdam, The Netherlands; 8 Institute of Clinical Chemistry and Clinical Pharmacology, University Hospital Bonn, Bonn, Germany; 9 Department of Neuromuscular Disorders, UCL Queen Square Institute of Neurology, London, UK; 10 NIHR Biomedical Research Centre, Wellcome Centre for Human Genetics, University of Oxford, Oxford, UK; 11 Manchester Centre for Genomic Medicine, St Mary’s Hospital, Health Innovation Manchester, Manchester University Foundation NHS Trust, Manchester, UK; 12 Division of Evolution, Infection and Genomics, School of Biological Sciences, Faculty of Biology, Medicine and Health, The University of Manchester, Manchester, M13 9PL UK; 13 Division of Neurology, Division of Genetics, Ann & Robert H. Lurie Children’s Hospital of Chicago, Chicago, USA; 14 GeneDx, Gaithersburg, MD 20877 USA; 15 Department of Biological Science, Dartmouth College, Hanover, NH USA; 16 Medical Genomics Research Department, King Abdullah International Medical Research Center (KAIMRC), King Saud Bin Abdulaziz University for Health Sciences, Ministry of National Guard Health Affairs, Riyadh, Saudi Arabia; 17 Department of Life Sciences, School of Science, University of Management and Technology (UMT), Lahore, Pakistan; 18 Neuroscience Centre, King Faisal Specialist Hospital and Research Centre (KFSHRC), MBC: 76, Riyadh, 11211 Saudi Arabia; 19 Molecular Oncology Department, King Faisal Specialist Hospital and Research Centre (KFSHRC), MBC: 03, Riyadh, 11211 Saudi Arabia; 20 Translational Genomics Department, Center for Genomics Medicine, King Faisal Specialist Hospital and Research Centre, MBC: 26, PO Box: 3354, Riyadh, 11211 Saudi Arabia; 21 Applied Genomics Technologies Institute, King Abdulaziz City for Science and Technology (KACST), Riyadh, Saudi Arabia; 22 CENTOGENE, GmbH, 18055 Rostock, Germany; 23 Clinical Genetics Department, Human Genetics and Genome Research Institute, National Research Centre, Cairo, Egypt; 24 Departments of Neurosciences and Pediatrics, Howard Hughes Medical Institute, University of California, Rady Children’s Institute for Genomic Medicine, San Diego, USA; 25 Centre de Biologie Structurale, CNRS, INSERM, Université de Montpellier, 34090 Montpellier, France; 26 ENCORE Expertise Center for Neurodevelopmental Disorders, Erasmus MC University Medical Center, Rotterdam, The Netherlands; 27 Discovery Unit, Department of Clinical Genetics, Erasmus MC University Medical Center, Rotterdam, The Netherlands

**Keywords:** Hereditary spastic paraplegia, Genetics, Neurology, AMFR, Cholesterol metabolism, Zebrafish disease modeling, Whole genome sequencing, Precision medicine, Statin

## Abstract

Hereditary spastic paraplegias (HSP) are rare, inherited neurodegenerative or neurodevelopmental disorders that mainly present with lower limb spasticity and muscle weakness due to motor neuron dysfunction. Whole genome sequencing identified bi-allelic truncating variants in *AMFR*, encoding a RING-H2 finger E3 ubiquitin ligase anchored at the membrane of the endoplasmic reticulum (ER), in two previously genetically unexplained HSP-affected siblings. Subsequently, international collaboration recognized additional HSP-affected individuals with similar bi-allelic truncating *AMFR* variants, resulting in a cohort of 20 individuals from 8 unrelated, consanguineous families. Variants segregated with a phenotype of mainly pure but also complex HSP consisting of global developmental delay, mild intellectual disability, motor dysfunction, and progressive spasticity. Patient-derived fibroblasts, neural stem cells (NSCs), and in vivo zebrafish modeling were used to investigate pathomechanisms, including initial preclinical therapy assessment. The absence of AMFR disturbs lipid homeostasis, causing lipid droplet accumulation in NSCs and patient-derived fibroblasts which is rescued upon AMFR re-expression. Electron microscopy indicates ER morphology alterations in the absence of AMFR. Similar findings are seen in *amfra-/-* zebrafish larvae, in addition to altered touch-evoked escape response and defects in motor neuron branching, phenocopying the HSP observed in patients. Interestingly, administration of FDA-approved statins improves touch-evoked escape response and motor neuron branching defects in *amfra-/-* zebrafish larvae, suggesting potential therapeutic implications. Our genetic and functional studies identify bi-allelic truncating variants in *AMFR* as a cause of a novel autosomal recessive HSP by altering lipid metabolism, which may potentially be therapeutically modulated using precision medicine with statins.

**Supplementary Information:**

The online version contains supplementary material available at 10.1007/s00401-023-02579-9.

## Introduction

Hereditary spastic paraplegia (HSP) is a group of rare, inherited neurodegenerative or neurodevelopmental disorders which mainly present with lower limb spasticity and muscle weakness due to dysfunction of motor neurons from the corticospinal tract [37]. HSPs are classified as pure or complex [13]. The former most common form manifests as isolated pyramidal signs, such as spasticity and hyperreflexia, which can be accompanied by sphincter dysfunction and deep sensory loss, while the latter presents with additional neurological features, including intellectual disability, cerebellar dysfunction, seizures, extrapyramidal signs, peripheral neuropathy, brain imaging abnormalities, and non-neurological features. The genetic landscape of HSP is complex, with currently more than 80 genetic HSP subtypes having been identified, involving all known modes of inheritance [8]. Most of these genes encode for proteins involved in intracellular trafficking, organelle distribution, axonal transport, myelination, mitochondrial function, and lipid metabolism, with dysfunction in these processes causing axonal degeneration of the longest descending motor fibers of the corticospinal tract leading to HSP.

Next-generation sequencing diagnostics, including gene panels and whole exome sequencing (WES), are key in HSP diagnostic, but only have a diagnostic yield of 30–60% [2, 28], indicating that there are still unknown causes of HSP that are missed. Among the emerging pathways leading to novel types of HSP are alterations in lipid metabolism [6]. Here, we identify bi-allelic loss-of-function of the autocrine motility factor receptor (*AMFR*, alias *GP78*), encoding a RING-H2 finger E3 ubiquitin ligase anchored at the endoplasmic reticulum (ER) membrane [9], as a new cause of HSP by altering lipid metabolism. Modulation of this metabolism by statins leads to restoration of phenotypes observed in zebrafish, potentially indicating a druggable target for precision medicine for this newly defined genetic disorder.

## Methods

### Patients

We studied 20 individuals affected by HSP from 8 families and provide their full clinical details in Table S1. All affected probands were investigated by their referring physicians and genetic analyses were performed in a diagnostic setting. For Erasmus MC, genome-wide investigations in a diagnostic setting were IRB-approved (METC-2012–387). Probands or their legal guardians gave informed consent for genomic investigations and publication of their anonymized data, including photographs and videos, in accordance with the Declaration of Helsinki.

### Genetic analysis

Whole Genome Sequencing (WGS), WES, or targeted Sanger sequencing was performed for probands and their tested family members. Full details of data generation, and computational analysis including protein modeling are provided in the **Supplementary Methods** (**Supplementary Appendix 1**).

### Functional studies

We performed functional studies using 1) CRISPR-Cas9 engineered embryonic stem cells (ESCs) differentiated to neural stem cells (NSCs); 2) patient-derived fibroblasts from Family 1 (IRB-approval MEC-2017–341) and Family 8 (obtained in a diagnostic setting); and 3) up to 5 day post-fertilization (dpf) old larvae from a generated zebrafish *amfra* knockout model. Full experimental details for functional studies are given in the Supplementary Methods (Supplementary Appendix 1).

### Statistical analysis

Data are provided as means or medians and standard errors of the mean (SEM) or standard deviation (SD) as indicated. Also indicated are statistical tests used to determine statistical significance and methods for multiple comparisons corrections. *p* < 0.05 was considered significant. Asterisks indicate significance levels (*p < 0.05; **p < 0.01; ***p < 0.001; ns = not significant).

## Results

### Loss-of-function AMFR variants in 20 individuals with autosomal recessive hereditary spastic paraplegia

**Fig. 1:**
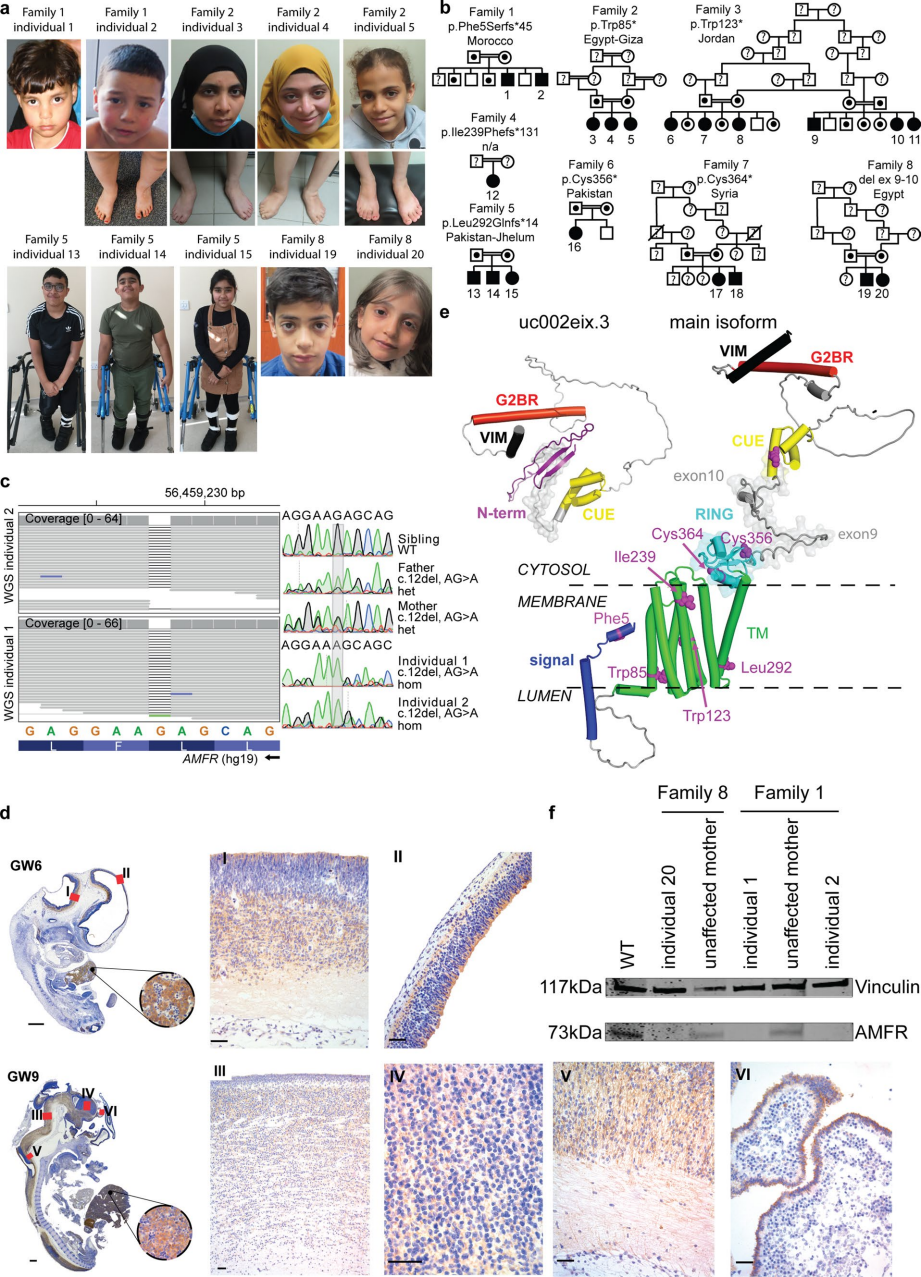
Homozygous *AMFR* loss-of-function variants in 20 individuals affected by HSP. **a** Clinical photographs of affected individuals at various ages. No major dysmorphic features are observed. **b** Family pedigrees of ascertained individuals, with familial *AMFR* variant and ethnic origin of families indicated. HSP-affected individuals with bi-allelic *AMFR* variants are indicated with black-filled symbols and numbered. Unaffected, heterozygous individuals are marked with a black dot. Unaffected individuals with wild-type alleles are represented by the non-filled symbols, whereas unaffected individuals of which no DNA was available for genotyping are marked with a question mark. Consanguineous parents are indicated with a double connection line. Males are squares; females are circles. **c** Genetic investigations in Family 1. Left panel shows aligned WGS reads from individual 1 and individual 2 in the IGV genome browser (left to right is from the centromeric to telomeric direction on chromosome 16q12.2), identifying a homozygous chr16(GRCh37):g56459228del in the first exon of *AMFR*. Right panel shows Sanger sequencing chromatograms (in the same orientation as the IGV genome browser view) from both affected homozygous individuals 1 and 2, both unaffected parents that are heterozygous for the deletion, and the unaffected, third oldest brother that is homozygous for the wild-type allele. Full segregation of the single-nucleotide deletion with the HSP phenotype in the Family 1 is shown in Supplementary Fig. 2. **d** Immunohistochemistry detecting AMFR in human fetuses at gestational week (GW) 6 and GW9. To characterize AMFR expression in various human tissues, we performed immunohistochemistry of human fetuses during the first trimester of pregnancy, using a C-terminal epitope recognizing antibody. AMFR staining of hepatocytes was noticed in all fetuses at GW6 and GW9. Central nervous system staining includes pale labeling of neuropil in the proliferative neuroepithelium of the hypothalamic, cortical, mesencephalic, and thalamic region (I, II, III and IV), as well as the marginal zone of the spinal cord (V) and cuboidal cells of choroid plexus (VI). **e** Structural protein model for the main AMFR isoform and the truncated isoform uc002eix.3, including the various variants identified in this study. Signal peptide is kept intact in the model to show the frameshift mutation, p.Phe5Serfs*45. The relative positions of AMFR domains are modified for clarity from the model predicted by AlphaFold2 (accession number AF-Q9UKV5-F1) [44]. Domains are color-coded. Other regions, including loops and unassigned structural elements, are shown in gray. Residues linked to patient variants are shown as pink sphere models. The region encoded by exon 9 and exon 10 is indicated by a transparent surface. The membrane and its cytosolic and luminal sides are outlined by dashed lines. Phobius (https://phobius.sbc.su.se) predictions were used to support the membrane location of AMFR regions. The main AMFR isoform contains an N-terminal signal sequence (residues 1–37), a multipass transmembrane domain (TMD) (residues 82–302), a cytosolic C-terminus which carries a Really Interesting New Gene (RING)-type zinc finger (residues 327–382), a coupling of ubiquitin conjugation to the ER degradation (CUE) domain (residues 457–497), a UBE2G2-binding region (G2BR) (residues 574–600), and a Valosin-containing protein (VCP)-interacting motif (VIM) (residues 622–640). These domains are connected by disordered regions, with residues 504–579 being compositionally biased toward polar and charged residues [43]. AMFR has an unusually open arrangement of the TMD helices, suggesting that they may form homo- or heterocomplexes. Nonetheless, the TMD appears dispensable for AMFR ligase activity [20]. The RING and G2BR domains interact with the E2 ubiquitin-conjugating enzyme UBE2G2, and the CUE domain recognizes ubiquitins on substrates. Association between the G2BR domain with UBE2G2 causes conformational alterations in UBE2G2, increasing affinity of UBE2G2 for AMFR. The VIM interacts with the AAA ATPase and segregase VCP/p97 [43]. Finally, the RING domain is tethered to the last TMD helix. Transcript uc002eix.3 (not annotated in NCBI) encodes a truncated form of AMFR (277 amino acids, 33 kDa), starting at an out of frame ATG in main transcript exon 7. This isoform lacks the TMD and RING domains N-terminal to the CUE domain, and therefore does not harbor E3 ligase activity. It has acquired 60 N-terminal residues without significant sequence identity to regions of known function for which AlphaFold2 predicts, with low confidence, the presence of a beta-sheet. **f** Western blotting analysis detecting AMFR and Vinculin in patient-derived fibroblasts from Family 1 and Family 8 and controls, showing the absence of the main AMFR protein isoform in patient cells. Full-length uncropped Western blot is given in Supplementary Fig. 4c

We encountered two siblings, born to consanguineous parents after uneventful pregnancies, who first came to medical attention around the age of 2 years due to developmental delay that developed progressively into lower limb spasticity (Fig. 1a, b**, **Table 1**, **Supplementary Table 1). At age 17 years, individual 1 had a spastic gait and required a wheelchair for longer distances. Individual 2, at age 10 years, had spasticity, a mild intellectual disability, and follows special education. Brain MRI showed nonspecific T2 hyperintensities in the left basal ganglia of individual 1 and was normal in individual 2 (Supplementary Fig. 1). No major dysmorphic features were noticed (Fig. 1a). Metabolic investigations in urine and serum were unremarkable, including FGF21 and cholesterol levels (Supplementary Fig. 2a). Prior extensive genetic investigations in both siblings including SNP arrays, HSP gene panels and trio WES, failed to identify a disease-explaining genetic variant. Additionally, re-analysis of exome data focusing on SNP-array derived runs of homozygosity (ROH), shared between both individuals but absent in four unaffected siblings was inconclusive. Both siblings were therefore included in a clinical WGS implementation project at Erasmus MC. Analysis focusing on all protein-coding genes did not identify a known disease-causing variant, but identified in both siblings a homozygous single-nucleotide deletion (chr16(GRCh37):g.56459228del) in *AMFR* exon 1, causing a p.Phe5Serfs*45 variant, which was absent in gnomAD [19]. *AMFR* is located within one of the shared ROHs. In agreement, segregation analysis confirmed that only both affected siblings were homozygous for this variant (Fig. 1c, Supplementary Fig. 2b). Re-analysis of the generated exomes showed that the variant was only present in five reads in one exome and, therefore, not previously called by the clinical analysis pipeline (Supplementary Fig. 2c). In agreement, coverage of the mutation site in *AMFR* exon 1 in clinical WES samples was poor compared to the average coverage in WGS samples (Supplementary Fig. 2d), explaining the previous failed detection of the variant. Subsequently, via GeneMatcher [38] and our international collaboration network, we identified seven additional families, resulting in a total cohort of 20 affected individuals with bi-allelic, protein truncating *AMFR* variants segregating in these families (Fig. 1a, b**, **Supplementary Fig. 3, Table1). All variants are absent from gnomAD (except c.254G > A, p.Trp85*, found once in heterozygosity among 76,036 genomes), and no other frameshift or stop gain variants are found in a homozygous state in gnomAD [19], Exome Variant Server [7], GME variome [35], or Iranome database [10]. Variants were predicted by MutationTaster [34] as disease causing, had high CADD scores, and involved conserved residues (Supplementary Table 2). Most individuals had a predominantly pure HSP. Mild intellectual disability (*n* = 4) or learning problems (*n* = 5) were observed in nine individuals. Two individuals had fever-induced seizures and four individuals had epilepsy. Other than a thin corpus callosum in five individuals, brain MRIs did not reveal major abnormalities. No major dysmorphic features were observed (Table 1**, **Supplementary Table 1, Supplementary Movie 1). Together, these clinical and genetic data from 20 individuals with HSP indicate *AMFR* as a likely HSP causing gene.

**Table 1:** Main clinical features of the cohort of 20 individuals with bi-allelic, loss-of-function *AMFR* variants. See the extended Supplementary Table 1 for additional information

	Phenotype	Percentage (%)	Total
Sex	Male	35	7/20
	Female	65	13/20
Consanguinity		100	20/20
Affected parents		0	0/20
Disease onset < 3 years		100	19/19
Premature death		0	0/20
Pregnancy & birth	Uneventful	90	18/20
	Maternal hypertension	10	2/20
	Cesarian section	25	5/20
	IUGR	5.3	1/19
	Perinatal problems	5	1/20
Development & cognition	Motor delay	100	20/20
	Speech delay	0	0/20
	Mild intellectual disability	20	4/20
	Learning problems	25	5/20
	Behavioral concerns	15.8	3/19
	Regression	0	0/20
Neurology	Axial hypotonia	10	2/20
	Lower limb hyperreflexia	100	20/20
	Spastic paraplegia	100	20/20
	Bladder dysfunction	0	0/19
	Ataxia	0	0/19
	Epilepsy	20	4/20
	Fever-induced seizures	10	2/20
	Thin corpus callosum	55.6	5/9
	Microcephaly	6.3	1/16
Major dysmorphism		0	0/20

### AMFR encodes a widely expressed E3 ubiquitin ligase which expression is disrupted by patient variants

*AMFR* is widely expressed (Fig. 1d**, **Supplementary Fig. 4a) and was initially described as an internalizing cell surface receptor for the autocrine motility factor (AMF)/phosphoglucose isomerase (PGI), a tumor secreted cytokine [27, 47]. AMFR also exhibits E3 ligase activity anchored at the ER membrane, where it mediates polyubiquitination of diverse substrates [9], including the cholesterol metabolism regulatory proteins 3-hydroxy-3-methylglutaryl-CoA reductase (HMGCR) and INSIG-1, in a process called ER-associated degradation (ERAD) [18, 40].

The UCSC genome browser [16] shows 4 relevant *AMFR* transcripts, of which NM_001144.6 (NCBI isoform c) (Supplementary Fig. 4b) is referred to herein as the main transcript. It encodes a 643-amino acid protein with a molecular weight of 73 kilodaltons (kDa) comprising several functional domains (Fig. 1e). All variants are predicted to result in a premature stop of the main isoform and isoform d, and should therefore evoke nonsense mediated decay (NMD). Western blotting analysis of fibroblasts from Family 1 (p.Phe5Ser*fs) and Family 8 (exon 9–10 deletion causing p.Asn362Lysfs*22), indeed, showed complete absence of the ~ 73 kD main AMFR isoform in affected individuals and reduced expression in heterozygous parents, without clear evidence for other truncated proteins or isoforms (Fig. 1f**, **Supplementary Fig. 4b,** c**)). Surprisingly, diagnostic RNA-seq of fibroblasts harboring p.Phe5Ser*fs did not show evidence of NMD (Supplementary Fig. 4d). Possibly, given the potential expression of multiple isoforms from the *AMFR* locus, NMD might be escaped as found for other loci [12, 26]. Despite this, if they would still be expressed at the protein level, the severely truncated variants p.Phe5Ser*fs, p.Trp85*, p.Trp123*, p.Ile239fs, p.Leu292Glnfs*14, p.Cys356Ter, and p.Cys364* affect all key domains in both the main isoform and isoform d (Fig. 1e**, **Supplementary Fig. 4e). Given that the TMD and C-terminal domains are required for E3 ubiquitin ligase activity of AMFR at the ER, these mutations will impair ERAD. Likewise, the exon 9–10 deletion will result in a non-functional protein, as besides truncating the RING domain of the main isoform, and of isoform d and e, the variant results in a premature stop in exon 11 (p.Asn362Lysfs*22), disturbing the CUE domain and eliminating the C-terminal G2BR and VIM domains.

Contrary, none of these variants, except exon 9–10 deletion, would affect the uc002eix.3 isoform, that lacks the TMD and RING domains and thus lacks ERAD activity. Also, p.Phe5Ser*fs and p.Trp85* would not affect isoform e, which lacks the first 95 N-terminal residues found in the main transcript. We cannot exclude that residual AMFR function might be present in non-investigated cell types of affected individuals with p.Phe5Ser*fs and p.Trp85* if isoform e is expressed to sufficient levels. However, a significant role of these variants seems unlikely as isoform e is likely unstable and mislocalized given the N-terminal signal peptide and TMD truncation. Also, as all identified individuals have a shared clinical phenotype, this argues for a common disease mechanism, which is not prevented by the potential presence of the truncated, non-membrane associated uc002eix.3 isoform. Together, these genetic and protein modeling data strongly argue that the mechanism of this novel disorder is driven by ERAD dysfunction of membrane bound AMFR, mediated by the longest isoforms.

### Human AMFR knockout neural stem cells show alterations in lipid metabolism

**Fig. 2:**
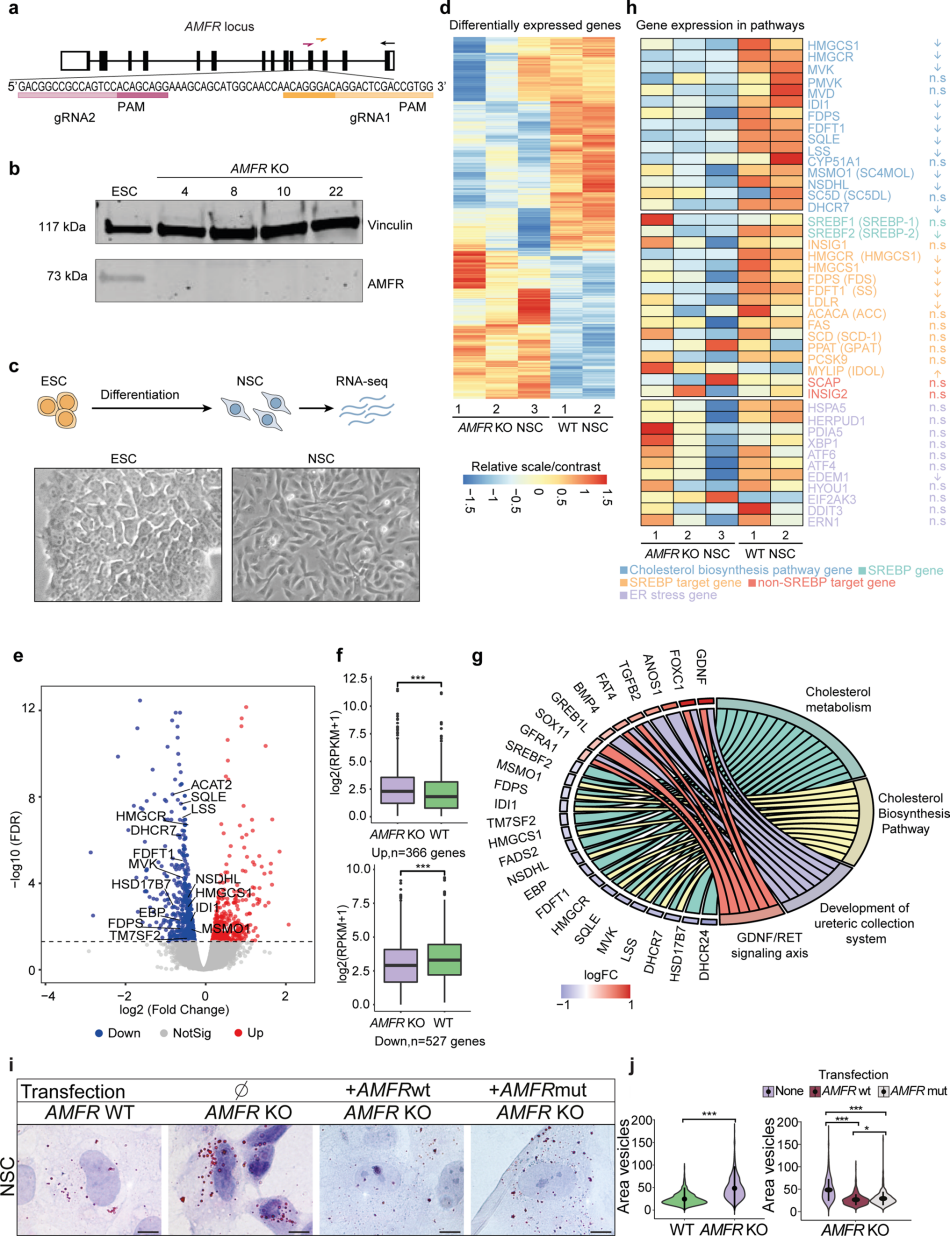
*AMFR* knockout neural stem cells show defects in cholesterol metabolism. **a** Schematic representation of the *AMFR* gene at chromosome 16q12.2. Filled boxes represent exons. Zoom-in shows the target sequence and gRNAs used to generate the *AMFR* KO ESCs. **b** Western blotting detecting AMFR (117 kDA) in wild-type H9 ESCs and *AMFR* KO ESC clones 4, 8, 10, and 22. Vinculin is used as a housekeeping control. Uncropped full-length Western blot is shown in Supplementary Fig. 5c. **c** Scheme showing the experimental outline and representative bright-field images of ESCs and differentiated NSCs. **d** Scaled heatmap depicting the RPKM gene expression from RNA-seq analysis of the 366 upregulated and 566 downregulated differentially expressed genes (FDR < 0.05) between wild type and *AMFR* KO NSCs. **e** Volcano plot showing log2 fold change and -log10(FDR) of the 366 upregulated and 566 downregulated differentially expressed genes between wild type and *AMFR* KO NSCs. Labels indicate selected genes. **f** Box plots showing the RNA-seq gene expression levels (log2(RPKM + 1) for the 366 upregulated and 566 downregulated genes in wild type and *AMFR* KO NSCs. Boxes represent the interquartile range (IQR); lines represent the median; whiskers extend to 1.5 × the IQR; dots represent outliers. (Wilcoxon test, *** *p *< 0.001). **g** GOChord plot of enrichment analysis, with the top-2 upregulated and downregulated terms from Wiki pathways, respectively. The left side of the GOChord diagram represents logFC, and the right side represents different terms of enrichment. The connecting bands indicate the corresponding pathways for each gene. **h** Scaled heatmap showing the RPKM gene expression levels for selected cholesterol biosynthesis pathway genes (blue), SREBP-1 and SREBP-2 (green), SREBP target genes (orange), non-SREBP target genes involved in cholesterol metabolism (red), and genes involved in ER stress response (purple). **i** Representative images of ORO staining detecting lipid droplets in wild type and *AMFR* KO NSCs, and in *AMFR* KO NSCs transfected with plasmids expressing wild-type AMFR or a RING mutant AMFR. Scale bars = 10 µm. **j** Quantification of the lipid droplet size from ORO staining from i). Violin plots showing the area of the quantified droplets in pixels and the distribution of the data. Data in the right plot are obtained from 2 wild-type NSCs (green colored) and 3 *AMFR* KO NSCs (purple colored) (biological replicates), cultured and stained each in two technical replicates, assessing n ≥ 400 droplets for each sample. Data in the left plot show the same quantification of the rescue experiments, where KO NSCs were transfected with a plasmid expressing wild type AMFR (brown) or a RING mutant AMFR (gray). Black circles, median; black line, SD (Kruskal–Wallis test for WT vs KO NSCs, Dunn’s Multiple Comparison test for the rescue experiment, *p < 0.05; ***p < 0.001)

For in vitro disease modeling in a patient-independent genetic background, we generated ESCs with a knockout in *AMFR* exon 4, allowing the comparison to isogenic wild-type controls (Supplementary Fig. 6a). We obtained multiple clones with compound heterozygous protein truncating indels that fully abolished the expression of the main AMFR isoform (Fig. 2a, b**, **Supplementary Fig. 5a, b). Interestingly, knockout ESCs showed an upregulation of the ~ 33 kDa band that was weakly expressed in parental ESCs (Supplementary Fig. 5c) and which likely corresponds to the truncated AMFR from transcript uc002eix.3 lacking ERAD activity, as confirmed at the cDNA level (Supplementary Fig. 5d, e).

For the remainder, we focused on knockout clones 4, 8, and 22 (Supplementary Fig. 5b, c) and refer to those as *AMFR* KO 1, 2, and 3. All *AMFR* KO ESCs showed normal morphology (Fig. 2c), growth, and pluripotency marker expression indistinguishable from wild type (Supplementary Fig. 6a). Upon NSC differentiation [31], all ESCs similarly acquired NSC morphology, downregulated pluripotency markers, and increased expression of the NSC marker PAX6 (Fig. 2c**, **Supplementary Fig. 6b, c). To identify possibly disease-relevant pathways in an unbiased manner using this neural cell type, we first assessed how AMFR loss would impact the global transcriptome by performing RNA-seq on wild-type parental and *AMFR* KO NSCs. RNA-seq showed high correlation between biological replicates and confirmed upregulation of NSC markers (Supplementary Fig. 6d, e). Differential gene expression analysis using edgeR [32] identified 366 genes that were significantly upregulated in *AMFR* KO NSCs compared to parental wild-type controls and 527 genes that were significantly downregulated (FDR < 0.05) (Fig. 2d-f). Gene ontology analysis using Enrichr [17] showed that upregulated genes were related to kidney development, cell migration, and spinal cord function, whereas downregulated genes were enriched for multiple terms related to cholesterol biosynthesis and cholesterol metabolism (Supplementary Fig. 6f, Supplementary Table 4). Further gene set enrichment analysis (GSEA) (Supplementary Fig. 6 g) and pathway analysis of genes related to cholesterol biosynthesis confirmed the general trend that expression of these genes was lower in *AMFR* KO NSCs compared to wild type (Fig. 2g, h), although the absolute fold changes in gene expression levels were relatively modest (Fig. 2f), as confirmed by qRT-PCR validation of selected genes (Supplementary Fig. 6 h).

Previously, it was shown that liver-specific *Amfr* loss in mice results in reduced HMGCR and Insig-1/-2 degradation [21]. Whereas increased levels of HMGCR, a rate-limiting enzyme in cholesterol synthesis that catalyzes the reduction of HMG-CoA to mevalonate, are expected to increase cholesterol biosynthesis, increased Insig-1/-2 levels lead to repression of sterol regulatory-binding protein (SREBP) processing and results in reduced expression of lipogenic SREBP target genes. In agreement, SREBP target genes showed a tendency toward lower expression in *AMFR* KO NSCs (Fig. 2h). Whereas, in mouse liver, the net effect of HMGCR and Insig-1/-2 stabilization upon *Amfr* loss is reduced cholesterol synthesis with reduced lipid droplets (LDs) in hepatocytes [21] and no increased Insig-2 stabilization was found in muscle of whole-body *Amfr* KO [50], another whole-body *Amfr* KO mouse did not display significant stabilization of HMGCR, Insig-1, and consequential suppression of SREBP-1 in knockout liver cells of young mice, but found upregulation of Insig-2 and increased susceptibility to ER stress [49]. This led to ER stress-mediated hepatic steatosis and obesity upon aging, caused by SREBP-1 activation with increased LDs in hepatocytes [49]. Similarly, siRNA knockdown of AMFR human Huh7 cells also resulted in LD accumulation [45].

To investigate how AMFR loss in NSCs would affect cholesterol and lipid metabolism, we stained NSCs using Oil Red O (ORO). The absolute LD number (Supplementary Fig. 6i) and size were significantly enlarged in *AMFR* KO NSCs compared to wild type (Fig. 2i, j). To confirm that this was caused by the absence of AMFR, we performed rescue experiments by transfecting *AMFR* KO NSCs with either wild-type AMFR or an AMFR mutant carrying two missense variants (Cys356Gly and His361Ala) in the RING domain [22] that are expected to compromise the zinc-binding ability and RING domain stability but leave all other AMFR domains intact. Whereas rescue with wild-type AMFR restored LD size to levels similar to wild type, rescue with the RING mutant AMFR was partial, possibly indicating that this mutant when overexpressed still retains residual AMFR activity and behaves hypomorphic (Fig. 2i, j). The effects on LD numbers were less pronounced (Supplementary Fig. 6j). Assessment of ER stress response did not show clear differences between wild type and *AMFR* KO NSCs, also upon treatment with tunicamycin (Fig. 2h**, **Supplementary Fig. 6k, l).

Together, this suggests that AMFR loss in human NSCs, contrary to loss of Amfr in mouse liver, results in a net effect of increased cholesterol synthesis, possibly due to stabilization of HMGCR, resulting in increased LD size and a compensatory downregulation of the lipogenic gene expression program due to effects on SREBP target genes.

### Patient-derived fibroblasts show altered lipid droplets which can be restored by AMFR re-expression and display dilated ER morphology

**Fig. 3:**
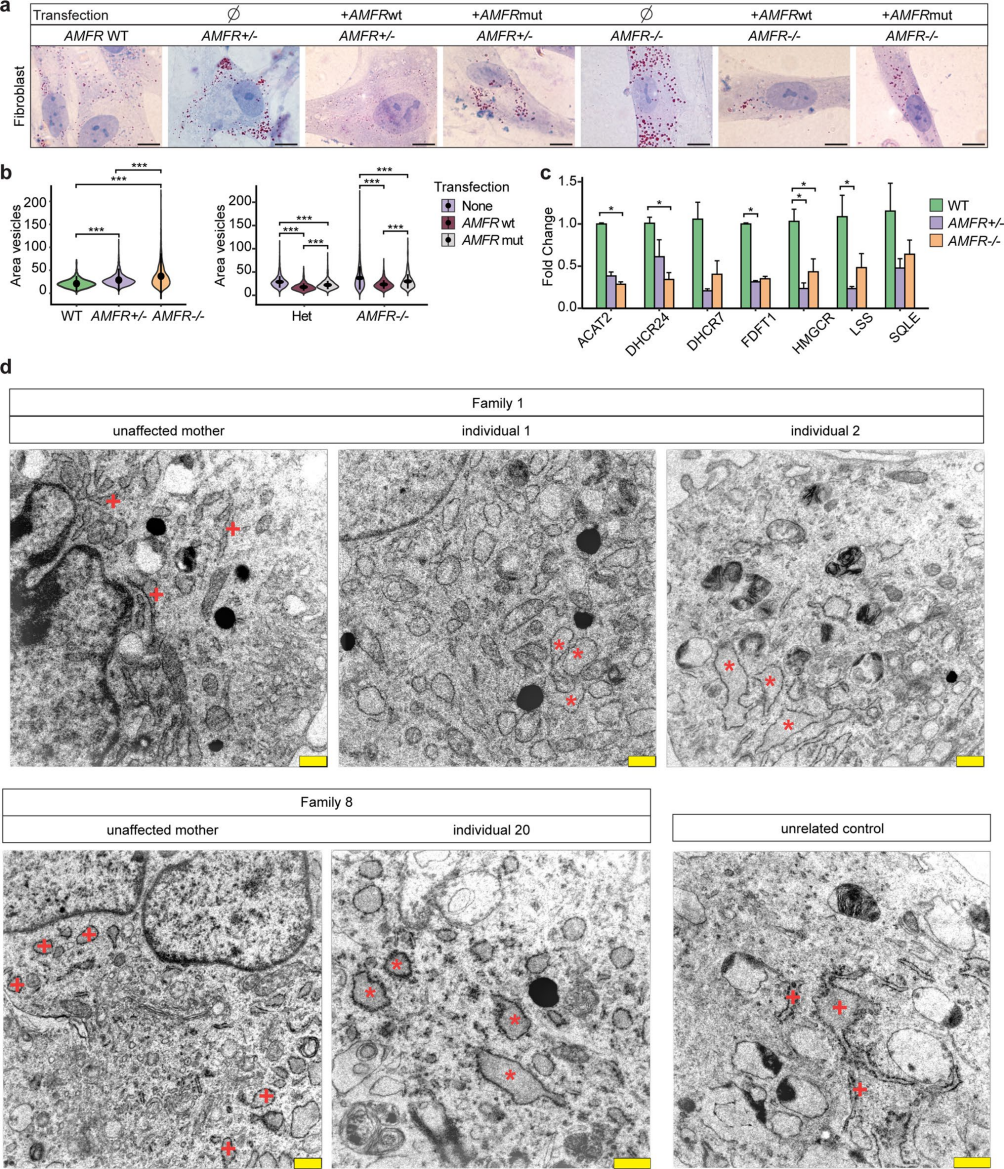
Patient-derived fibroblasts with *AMFR* loss-of-function variants show disturbed lipid metabolism and altered endoplasmic reticulum morphology **a** Representative images of ORO staining identifying lipids in unrelated wild type, heterozygous carrier (unaffected mothers from Family 1 and 8) and homozygous *AMFR* patient-derived fibroblasts from individuals 1, 2, and 20, and in the same fibroblasts transfected with plasmids expressing wild-type AMFR or RING mutant AMFR. Scale bars = 10 µm. **b** Left violin plots showing the size area of the quantified lipid droplets in pixels from ORO stained fibroblasts from a). Data from 2 wild-type fibroblasts (green colored), 2 heterozygous carrier *AMFR* fibroblasts (unaffected mothers of Family 1 and 8, purple colored) and 3 homozygous *AMFR* patient-derived fibroblasts (from individuals 1, 2, and 20, orange colored) (biological replicates), cultured and stained each in two technical replicates, assessing n ≥ 400 droplets for each sample. Black circle, median; black line, SD (Dunn’s Multiple Comparison test, ***p < 0.001). Right violin plot shows the same quantification from a), but then for heterozygous or homozygous *AMFR* fibroblasts transfected with plasmids expressing wild type AMFR or RING mutant AMFR, as indicated. **c** qRT-PCR expression analysis of genes involved in cholesterol metabolism in wild type (n = 2), heterozygous (*n* = 2, from unaffected mothers of Family 1 and 8), and homozygous (*n* = 3, from individuals 1, 2, and 20) *AMFR* patient-derived fibroblasts. Bar plot showing the mean fold change for the indicated genes compared to wild type, normalized for the housekeeping gene *TBP*. Each fibroblast line was cultured in two independent duplicates, and measured using two technical replicates and two independent experiments. Error bars represent SEM (Dunn’s Multiple Comparison test, *p < 0.05; **p < 0.01; ***p < 0.001). **d** Electron microscopy of cultured fibroblasts from individuals 1, 2, and 20, harboring homozygous truncating *AMFR* variants, as well as their respective heterozygous carrier mothers and an unrelated control. Affected individuals show an abundance of large vesicles indicative of lipid droplets as well as large, dilated rough endoplasmic reticulum (RER, red asterisks) as compared to the more compact RER seen in unaffected parents and an unrelated control (red + signs). Scale bars = 500 nm

To investigate if altered lipid metabolism is relevant in patient cells, we obtained fibroblasts from three affected individuals, their healthy heterozygous carrier mothers, and unrelated wild types. Culturing these fibroblasts under routine conditions, we observed that the median LD size in patient-derived cells was significantly larger compared to wild-type controls (Fig. 3a, b). This could be rescued by overexpression of wild-type AMFR, but only partially with the hypomorphic mutant AMFR (Fig. 3a, b). Interestingly, heterozygous *AMFR* carrier fibroblasts showed an intermediate phenotype, with significantly larger LDs compared to wild type, but also significantly smaller LDs compared to patient-derived fibroblasts (Fig. 3a, b). Also, this was amenable to rescue with wild-type AMFR, but only partially by the hypomorphic mutant AMFR (Fig. 3a, b). This argues that LD size correlates in a dose-dependent manner with the available quantity of functioning AMFR. No effect on the number of LDs per cell in fibroblasts was observed (Supplementary Fig. 7a).

In agreement with LD findings in fibroblasts and NSCs, and downregulation of cholesterol biosynthesis genes in *AMFR* KO NSCs, cholesterol metabolism genes in fibroblasts from affected individuals were significantly downregulated compared to controls, although differences in expression levels compared to heterozygous carrier parents were less pronounced (Fig. 3c). No consistent differences in ER stress gene expression were noticed (Supplementary Fig. 7b).

To further study LDs at ultrastructural level, we examined the same fibroblasts using electron microscopy (EM) (Fig. 3d). In addition to large vesicles reminiscent of LDs but not lysosomes (Supplementary Fig. 7c), patient samples displayed substantially dilated ER morphology that was not seen in parental and wild-type samples. Other organelles, including mitochondria and Golgi apparatus, did not show notable differences.

We conclude that loss-of-function variants in *AMFR* cause alterations of LDs and ER morphology in patient-derived fibroblasts.

### Zebrafish lacking Amfra show lipid accumulations, aberrant ER morphology, abnormal motor neuron branching, and aberrant touch-evoked escape response which can be rescued by statin treatment

**Fig. 4:**
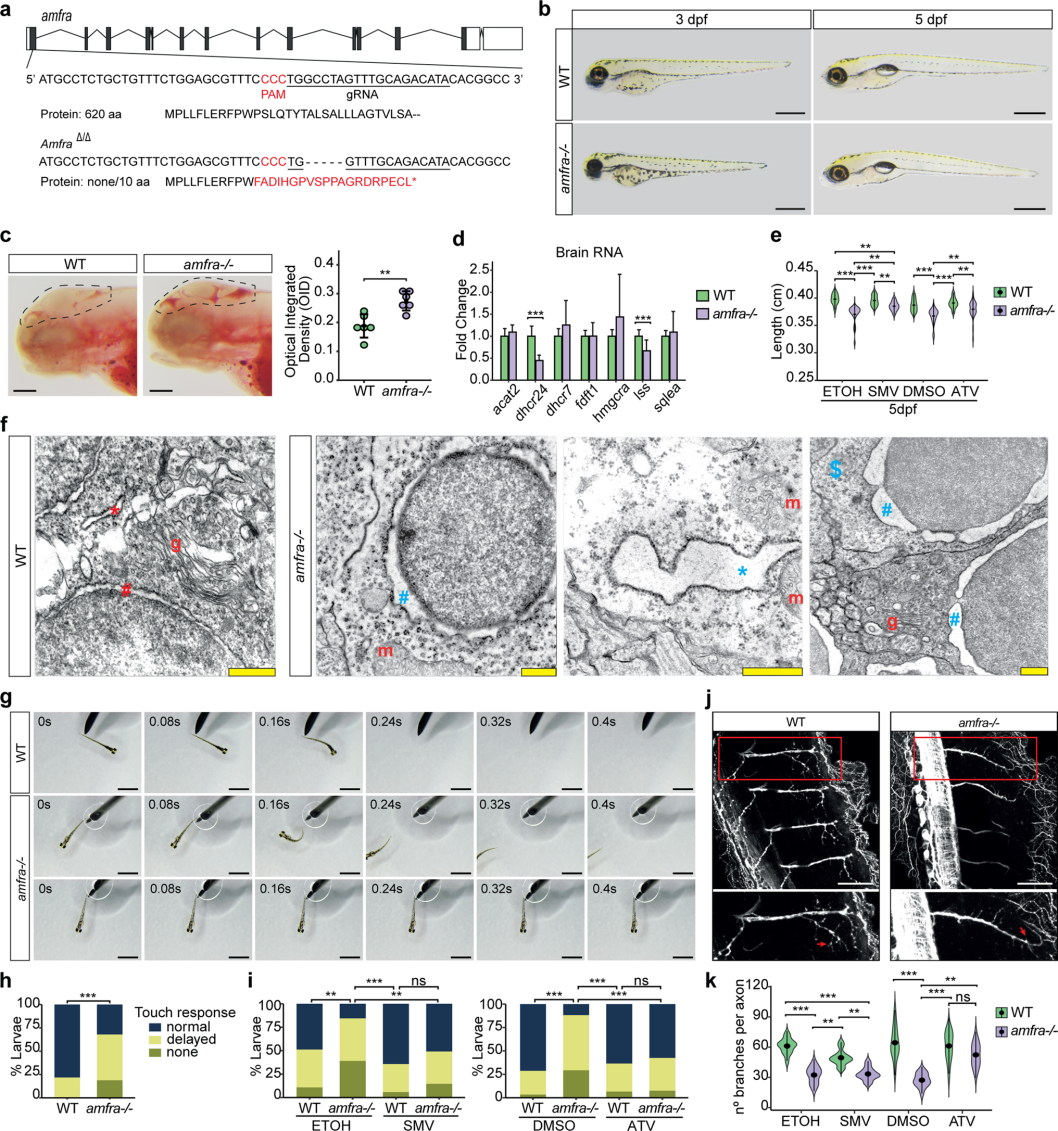
*amfra-/-* zebrafish larvae show alterations in lipid metabolism and endoplasmic reticulum morphology, abnormal touch-evoked escape response and motor neuron branching defects that can be rescued by statin treatment. **a** Schematic drawing of the *amfra* locus in zebrafish, the gRNA used and the generated mutation, causing a 5 bp frameshift in the first coding exon of *amfra*. Coding exons, black; non-coding exons, white. **b** Representative bright-field images of wild type and *amfra-/-* larvae at 3 dpf and 5 dpf. *amfra-/-* larvae appear morphologically similar to wild-type (WT) larvae at both developmental stages. Scale bars = 500 µm. **c** Representative images and quantification of ORO staining of 3 dpf larvae, dotted line indicating the region of interest (ROI) used for quantifications. Circles show individual values for each larva, *n* = 6 larvae per genotype (WT, green; *amfra-/-*, purple). Error bars represent SD (Kruskal–Wallis test, **p < 0.01). Scale bars = 100 µm. **d** qRT-PCR expression analysis for selected cholesterol metabolism genes, in brains of 5 dpf control and *amfra-/-* larvae (*n* = 10 brains per sample, 4 biological replicates for WT larvae and 5 biological replicates for *amfra-/-*, from 2 independent experiments, with each biological replicate measured in two technical replicates). Bar plot showing the mean fold change for the indicated genes compared to control larvae, normalized for the housekeeping gene *eef1a1*. WT, green. *amfra-/-*, purple. Error bars represent SD (Kruskal–Wallis test, ***p < 0.001). **e** Violin plot showing the length of wild type (WT) and *amfra-/-* larvae at 5 dpf. Larvae are either treated starting from 8 hpf onwards with simvastatin (SMV) or atorvastatin (ATV), or with their respective vehicle controls ethanol or DMSO. Whereas statin treatment does not influence the length of WT larvae, it significantly increases the length of *amfra-/-* larvae, partially rescuing the observed length deficit of *amfra-/-* larvae compared to WT. n > 20 per genotype and treatment group (Dunn’s Multiple Comparison test, **p < 0.01; ***p < 0.001). **f** Electron microscopy of brains from WT and *amfra-/-* larvae at 5 dpf. *amfra-/-* larvae show dilated rough endoplasmic reticulum decorated with ribosomes (blue *) and an expanded perinuclear space (blue #) as compared to controls (red * and #, respectively). Many cells in the *amfra-/-* samples have less densely stained cytoplasm (example indicated with a blue $). m = mitochondrion (normal appearing) and g = Golgi apparatus (normal appearing). Scale bars = 500 nm. **g** Representative bright-field images for the touch-evoked escape response (upper row: normal response; middle row: delayed response; bottom row: no response) of 3 dpf WT and *amfra-/-* larvae. Scale bars = 500 µm. **h** Quantification of the touch-evoked escape response in WT and *amfra-/-* larvae at 3 dpf, n > 45 per genotype, from 3 experimental replicates (Chi-square test, ***p < 0.001). **i** As H, but now for WT and *amfra-/-* larvae treated with simvastatin (SMV) or atorvastatin (ATV) and their respective vehicle controls, ETOH and DMSO (Chi-square test, **p < 0.01; ***p < 0.001; ns = not significant). **j** Immunostaining for acetylated tubulin in WT and *amfra-/-* embryos. Shown are max projections from z-stacks of 2 dpf embryos, acquired from lateral view of the middle of the trunk. The region indicated by the red rectangle is shown in enlargement in the insert below. *amfra-/-* embryos show reduced axon branching in ventral motor neurons in comparison to control (red arrow indicates a branching). Scale bars = 50 µm. **k **Violin plot showing the quantification of axon branching of ventral motor neurons in WT (green) and *amfra-/-* embryos (purple), treated with vehicle (ETOH or DMSO) or statin (SMV or ATV); *n* = 6 embryos per group; for each embryo, two axons were quantified. Black circle, median; black line, SD (Dunn’s Multiple Comparison test, **p < 0.01; ***p < 0.001; ns = not significant)

To model *AMFR* dysfunction in vivo, we generated a zebrafish model. Zebrafish carry two orthologues of *AMFR*, *amfra* and *amfrb*, of which only *amfra* shows detectable expression within 14 dpf (Supplementary Fig. 8a). Using CRISPR-Cas9, we obtained a mutant allele with a 5 bp frameshift deletion in *amfra* exon 1 (Fig. 4a**, **Supplementary Fig. 8b). Larvae homozygous for this allele (*amfra-/-*) are viable and show normal gross morphology (Fig. 4b), despite being significantly shorter at both 3 and 5 dpf compared to controls (Supplementary Fig. 8c). While a small but significant fraction of *amfra-/-* larvae fail to properly inflate their swim bladder by 5 dpf (Supplementary Fig. 8d), the majority do. Together, this indicates that the length discrepancy is unlikely to be due to a general delay in development.

Anticipating a possible lipid homeostasis dysregulation as found in human cells, we first investigated the distribution of lipids in *amfra-/-* larvae in the nervous system. At 3 dpf, *amfra-/-* larvae demonstrated a significantly higher ORO staining intensity throughout their brains compared to wild type (Fig. 4c**, **Supplementary Fig. 9a). qRT-PCR analysis assessing lipogenic gene expression at 5 dpf revealed significant downregulation of *lss* in only *amfra-/-* brains and reduction of *dhcr24* in whole *amfra-/-* larvae and extracted *amfra-/-* brains, but not in *amfra-/-* bodies (Fig. 4d**, **Supplementary Fig. 8e). To further assess cholesterol metabolism, we determined cholesterol and non-cholesterol sterol levels in *amfra-/-* and wild-type brain, bodies or whole larvae at 5 dpf. Assessing cholesterol corrected levels of the precursor lathosterol as endogenous surrogate marker of cholesterol synthesis rate [3], we found consistently a higher ratio of lathosterol to cholesterol in *amfra-/-* tissues compared to controls (Supplementary Fig. 9d, Supplementary Table 6). In agreement with increased synthesis, the ratio between campesterol to cholesterol, which reflects the sterol absorption rate [41], was lower in *amfra-/-* tissues (Supplementary Fig. 9e).

Similar to patient-derived fibroblasts, EM showed dilated ER morphology in *amfra-/-* brains, including expanded perinuclear spaces, and a prevalence of less densely stained cytoplasm, compared to wild type (Fig. 4f). Other organelles including mitochondria appeared normal, arguing against apoptosis.

Given these similarities between patient-derived cells and *amfra-/-* zebrafish, we next assessed published assays to study HSP in zebrafish [25], focusing on touch-evoked escape response [5] and motor neuron axon morphology [28, 30]. At 3 dpf, *amfra-/-* larvae showed a significantly reduced touch-evoked escape response, with more non-responding or delayed larvae compared to wild type (Fig. 4g, h**, **Supplementary Movie 2). Assessing the axon morphology of ventral motor neurons in 2 dpf embryos using acetylated tubulin immunostaining further revealed that *amfra-/-* embryos had significantly fewer branches per axon as compared to controls (Fig. 4j, k**, **Supplementary Movie 3). Both results are in agreement with previous findings in HSP zebrafish models [5, 28, 30], providing further evidence that AMFR dysfunction, as modeled in the *amfra-/-* larvae, underlies the observed patient phenotypes.

Given the observed disturbance of lipid homeostasis both in human and zebrafish models, and the previous findings that AMFR plays an important role in ERAD-mediated degradation of HMGCR [9], a key enzyme in cholesterol metabolism expected to be stabilized in the absence of AMFR [21], we next assessed whether inhibitors of HMGCR could positively modulate the phenotypes observed in zebrafish. An FDA-approved and clinically widely used class of HMGCR inhibitors are statins. Treatment of *amfra-/-* embryos from 8 hpf using simvastatin (SMV) and atorvastatin (ATV) led to a significant increase in length of *amfra-/-* larvae at 5 dpf (Fig. 4e) compared to vehicle-treated controls, without affecting wild types. At 3 dpf the same trend was observed, although effects were more marginal (Supplementary Fig. 8f). Assessing ORO staining intensity at 3 dpf did not show differences between statin and vehicle-treated control *amfra-/-* larvae (Supplementary Fig. 9b, c). In contrast, assessing touch-evoked escape response of 3 dpf *amfra-/-* larvae treated with either SMV or ATV showed a striking rescue of the behavior to levels indistinguishable from wild type (Fig. 4i). Finally, ATV but not SMV treatment fully corrected axon branching defects observed in *amfra-/-* larvae (Fig. 4k**, **Supplementary Fig. 8g, Supplementary Movie 4), with also the most pronounced effect on sterol ratios observed for ATV in brain cells (Supplementary Fig. 9d, Supplementary Table 5).

Together, this indicates that *amfra-/-* zebrafish phenocopy disease mechanisms observed in HSP that are caused by AMFR dysfunction in humans and that treatment with statins improves the observed phenotypes in this preclinical model, possibly pointing toward a road to personalized medicine for this newly defined disorder.

## Discussion

Here, we provide genetic and functional evidence that *AMFR* dysfunction in humans causes HSP by altering lipid homeostasis. *AMFR* adds to a number of identified HSP genes, where alterations in lipid metabolism are emerging as a common pathomechanism [6]. These include ERLIN1 and ERLIN2, multimeric ER-anchored proteins interacting with the SREPBP–Scap–Insig complex [1, 14, 28], and CYP7B1, an enzyme involved in cholesterol degradation [42]. Despite this clear evidence, how exactly altered cholesterol homeostasis contributes to axonal loss and neurodegeneration remains mysterious. Our findings, in addition to the previous studies [1, 14, 28], point to an important role of pathways converging on ER function as being relevant to long-term axonal wellness [39].

**Fig. 5:**
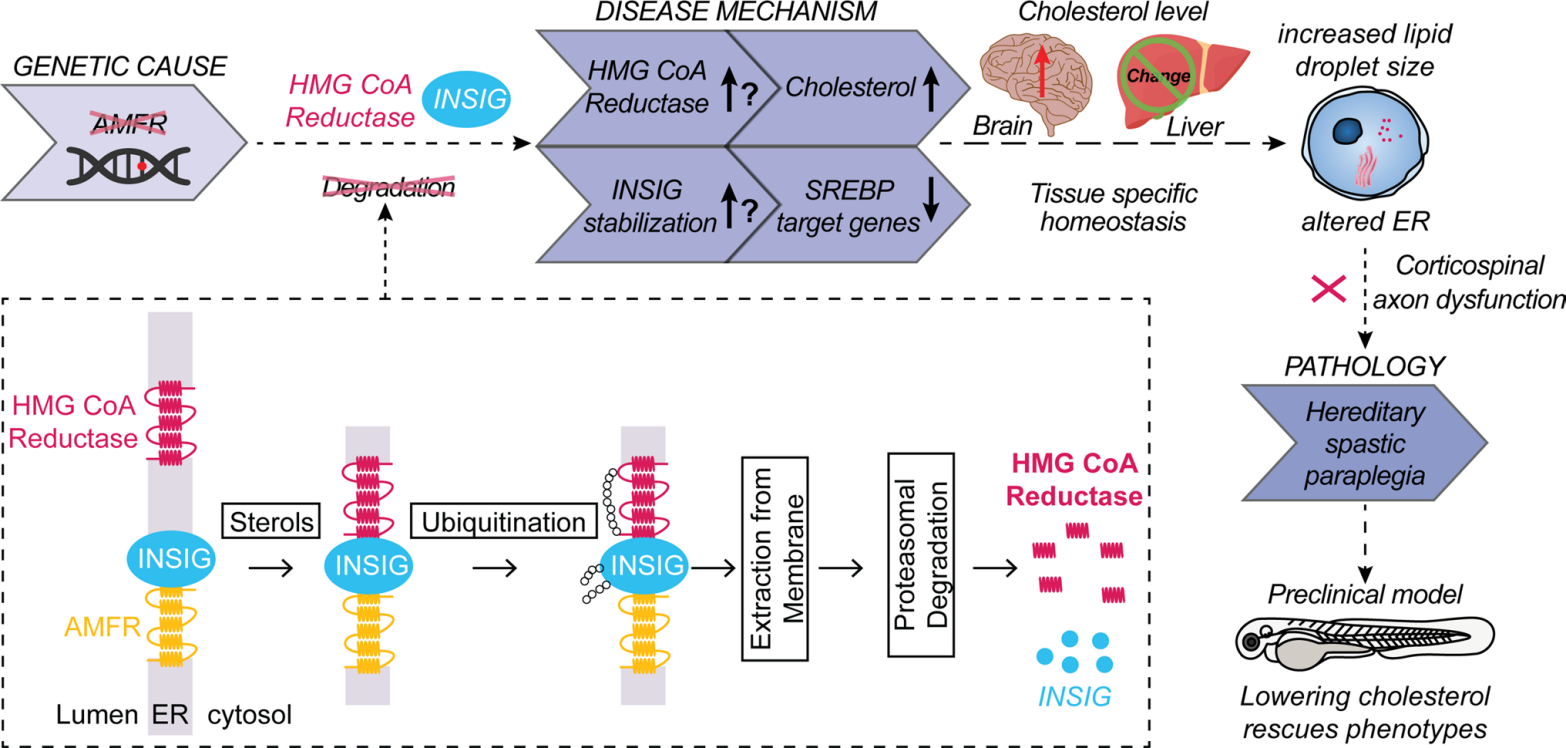
Hypothetical model describing the processes leading to HSP upon AMFR dysfunction. Upon loss of function of AMFR, degradation of HMGCR and INSIG proteins is likely blunted, resulting in an increased cholesterol synthesis rate upon HMGCR stabilization and a compensatory repression of SREBP processing causing reduced expression of lipogenic SREBP target genes upon stabilizing of INSIG proteins. The net effect of these opposing lipogenic and non-lipogenic processes is a disturbance in the balance of lipid and cholesterol homeostasis which is reflected by increased lipid droplet size in patient-derived fibroblasts and in human neural stem cells in the absence of AMFR. Possibly, cell-type-specific and species-specific differences in the balance between both opposite processes might occur, explaining the absence of increased cholesterol levels in patient serum and contradictory results in *Amfr* knockout mice. The increased lipid droplet size and concomitant alterations in the morphology of the endoplasmic reticulum then lead to neurotoxicity, causing dysfunction of the corticospinal tract due to an yet to be explored pathomechanism. Upon cholesterol lowering therapy in *amfra-/-* zebrafish larvae, this neurotoxicity is prevented, causing rescue of the observed phenotypes

Variants in Family 1 and 2 were only identified upon WGS and missed in clinical WES, possibly due to high GC content contributing to poor *AMFR* exon 1 coverage. This suggests that targeted investigations of *AMFR* exon 1 in unexplained HSP patients might help increase diagnostic yields. The mutation modeling, the disturbed lipid homeostasis in *AMFR* mutant cells, and rescue of the *amfra-/-* zebrafish upon treatment with HMGCR-targeting statins argue that the loss of AMFR’s ERAD function likely underlies the disease mechanism of this new disorder, leading to a disturbance in the balance of lipid and cholesterol homeostasis in cells without AMFR (Fig. 5). Possibly, also ERAD-independent AMFR roles might contribute (Supplementary Note).

Previous studies on two generated *Amfr* mouse models have reached conflicting conclusions regarding the role of Amfr in cholesterol metabolism and ER stress in vivo [21, 49], (Supplementary Note). In patient-derived fibroblasts, and *AMFR* KO NSCs, no clear evidence for increased ER stress was found (Fig. 2h**, **Supplementary Fig. 2i, j, Supplementary Fig. 7b). Also, the lipogenic profile in serum of individuals 1 and 2 was normal, including FGF21 levels (Supplementary Fig. 2a). Enlarged LDs were observed in patient-derived fibroblasts and *AMFR* KO NSCs, arguing for increased cholesterol synthesis and a compensatory downregulation of SREBP target genes as detected by RNA-seq. Given the contrasting results in mice between different models and tissues, it seems likely that tissue-specific differences in the balance between lipogenic and non-lipogenic effects of AMFR loss might explain the absence of an altered lipogenic profile in serum of patients (mainly reflecting liver cholesterol synthesis) and the increased size of LDs in neuronal cells. Further distinguishing these tissue-specific differences is warranted, especially since modulation of AMFR levels has been proposed to have therapeutic effects for epilepsy [36], asthma [48], and metabolic syndrome and obesity [21], but these might thus cause neural dysfunction if not applied in a tissue-specific manner.

Peculiarly, upon ERLIN1 and ERLIN2 dysfunction, a similar LD increase was observed [14], thought to be caused by the lack of SREBP ER retention, leading to upregulation of lipogenic genes. This might be further increased by the ability of ERLIN2 to promote ERAD of HMGCR [15], for which AMFR is required to bridge ERLIN2 and INSIG. Also, upon ERLIN knockdown, proteasomal degradation of INSIG-1 is increased [14]. Thus, although at first the effects of AMFR and ERLIN dysfunction seem opposite (e.g., effect on SREBP target genes, INSIG-1 stabilization), their downstream effects both lead to increased LDs, and likely similarly affect motor neuron function. Potentially, lipid accumulation subsequently impairs ER architecture and function [4]. It will be interesting to explore whether similar ER morphologic changes as observed upon *AMFR* dysfunction are also seen in cells upon ERLIN1/2 loss, which might provide insights in what is causing the motor neuron damage.

Despite the overwhelming evidence that AMFR dysfunction causes HSP from human genetic and zebrafish studies presented here, so far, no signs of HSP are reported in *Amfr* knockout mice [21, 49]. It will be interesting to fully investigate locomotion, behavior, and motor neurons in these mice, as it remains possible that species-specific differences only cause subclinical phenotypes in mice that remained unnoticed or only become evident upon aging, as also many other HSP mouse models have failed to show gross abnormalities [11]. Interestingly, metabolic parameters and expression of cholesterol biosynthesis genes in mice with a heterozygous, liver-specific *Amfr* knockout showed intermediate phenotypes, compared to homozygous and wild-type animals [21]. Potentially, this is reminiscent of our observations in parental fibroblasts, which also show intermediates LD size. As heterozygous carriers are not affected, this might indicate that only above a certain level of lipid accumulation, a threshold is reached causing early onset neuropathology. Long-term follow up of heterozygous individuals could be considered, to uncover potential signs of accelerated neurodegeneration upon aging.

An intriguing finding is the observations that treatments with FDA-approved HMG-CoA reductase inhibitors, simvastatin and atorvastatin, can rescue phenotypes observed in *amfra-/-* zebrafish, pointing toward a potential route for precision medicine for this newly defined disorder. Both simvastatin and atorvastatin are highly lipophilic, allowing them to cross the blood–brain barrier and translating into decreased cholesterol levels locally in the brain [29], which is especially relevant, since most cholesterol is locally synthesized in the central nervous system [29]. Therefore, statins potentially could also reduce cholesterol in neural cells of *AMFR* patients, even if due to tissue-specific effects of AMFR loss no increased cholesterol levels are detected in blood. In spastic paraplegia caused by *CYP7B1* mutations, two short-term phase II clinical trials have shown that atorvastatin can reduce levels of oxysterols which lead to neurotoxicity in that disorder [24, 33]. Simvastatin can also reduce dehydrocholesterol levels in Smith–Lemli–Opitz syndrome [46]. Since long-term statin therapy is safe and well tolerated, even when treating children for 20 years [23], this might promise that statin treatment of the HSP disorder that we identify here, could also provide positive effects in humans as observed in zebrafish, although future clinical trials are required to determine the value of this potential therapeutic direction.

## Supplementary Information

Below is the link to the electronic supplementary material.

Supplementary file1 (DOCX 9283 KB)

Supplementary file2 (MP4 13418 KB)

Supplementary file3 (MP4 17316 KB)

Supplementary file4 (AVI 80496 KB)

Supplementary file5 (AVI 71811 KB)

Supplementary file6 (XLSX 34 KB)

Supplementary file7 (XLSX 14 KB)

Supplementary file8 (XLS 4402 KB)

Supplementary file9 (XLSX 479 KB)

Supplementary file10 (XLSX 28 KB)

Supplementary file11 (XLSX 13 KB)

## Data Availability

RNA-Seq of NSCs is publicly available through the National Center for Biotechnology Information (NCBI) Gene Expression Omnibus (GEO) under accession number GSE202141. Genome sequencing data for family 5 is available in the National Genomic Research Library from the 100,000 Genomes Project for which researchers can apply for access at Genomics England. For the other families, due to privacy regulations and given consent under which patients were recruited, raw patient RNA-seq data and genomic sequencing data cannot be made available. Codes used for data analysis are available via GitHub: https://github.com/barakatlab/AMFR_paper.git.
