# Supplementary material for: AMFR dysfunction causes autosomal recessive spastic paraplegia in human that is amenable to statin treatment in a preclinical model
Source: Acta Neuropathol. 2023 Apr 29;146(2):353–68. doi: 10.1007/s00401-023-02579-9 (PMC10328903; doi:10.1007/s00401-023-02579-9)
Supplement: Supplementary file 1 — Supplementary file1 (DOCX 9283 KB) [file 401_2023_2579_MOESM1_ESM.docx]

Supplementary Appendix

AMFR dysfunction causes autosomal recessive spastic paraplegia in human that is amenable to statin treatment in a preclinical model

Table of Contents

[List of authors and affiliations 4](#_Toc123500467)

[Author contributions 5](#_Toc123500468)

[Supplementary Methods: 6](#_Toc123500469)

[Patient Genetic investigations 6](#_Toc123500470)

[Structural protein modeling 7](#_Toc123500471)

[Fibroblast cell culture 7](#_Toc123500472)

[Human fetal samples and immunohistochemistry 8](#_Toc123500473)

[Genome engineering of human embryonic stem cells 8](#_Toc123500474)

[Neural stem cell differentiation 8](#_Toc123500475)

[qRT-PCR analysis 9](#_Toc123500476)

[RNA-sequencing and data analysis 9](#_Toc123500477)

[Western blotting 10](#_Toc123500478)

[Oil Red O staining in human cells 10](#_Toc123500479)

[Electron microscopy 11](#_Toc123500480)

[Immunocytochemistry in human cells 11](#_Toc123500481)

[Zebrafish husbandry 11](#_Toc123500482)

[CRISPR-Cas9 Genome editing in zebrafish 12](#_Toc123500483)

[Oil Red O staining in zebrafish 12](#_Toc123500484)

[Immunohistochemistry on zebrafish embryos 13](#_Toc123500485)

[Behavioral analysis 13](#_Toc123500486)

[Sterol analysis 13](#_Toc123500487)

[Statistical analysis and data presentation 14](#_Toc123500488)

[Data Availability 14](#_Toc123500489)

[Supplementary Notes 15](#_Toc123500490)

[Possible ERAD independent pathomechanisms of AMFR dysfunction 15](#_Toc123500491)

[Differences in findings from Amfr knockout mouse models 15](#_Toc123500492)

[Supplementary Figures and Legends: 16](#_Toc123500493)

[Supplementary Figure 1: MRI brain imaging in individuals with AMFR loss of function variants. 16](#_Toc123500494)

[Supplementary Figure 2: Metabolic and genetic investigations in Family 1. 19](#_Toc123500495)

[Supplementary Figure 3: Genetic investigations in families 2-8 21](#_Toc123500496)

[Supplementary Figure 4: AMFR is widely expressed and encodes potentially multiple isoforms of which the main isoform is predominant. 23](#_Toc123500497)

[Supplementary Figure 5: Generation of AMFR knockout embryonic stem cells 25](#_Toc123500498)

[Supplementary Figure 6: AMFR knockout neural stem cells show alterations of cholesterol homeostasis. 27](#_Toc123500499)

[Supplementary Figure 7: Additional analysis of patient-derived fibroblasts. 29](#_Toc123500500)

[Supplementary Figure 8: Generation and characterization of the amfra-/- zebrafish model. 32](#_Toc123500501)

[Supplementary Figure 9: Oil Red O staining in zebrafish larvae. 33](#_Toc123500502)

[Overview of separately supplied files: 35](#_Toc123500503)

[Supplementary Table 1-6 and Supplementary Movie 1-4 35](#_Toc123500504)

[Supplementary References: 36](#_Toc123500505)

# List of authors and affiliations

Ruizhi Deng*^1,2^, Eva Medico-Salsench*^1^, Anita Nikoncuk*^1^, Reshmi Ramakrishnan^3^, Kristina Lanko^1^, Nikolas A. Kühn^4^, Herma C van der Linde^1^, Sarah Lor-Zade^1^, Fatimah Albuainain^1^, Yuwei Shi^1^, Soheil Yousefi^1,2^, Ivan Capo^5^, Evita Medici van den Herik^6^, Marjon van Slegtenhorst^1^, Rick van Minkelen^1^, Geert Geeven^1,2^, Monique T. Mulder^7^, George J.G. Ruijter^1^, Dieter Lütjohann^8^, Edwin H. Jacobs^1^, Henry Houlden^9^, Alistair T. Pagnamenta^10^, Kay Metcalfe^11,12^, Adam Jackson^11,12^, Siddharth Banka^11,12^, Lenika De Simone^13^, Abigail Schwaede^13^, Nancy Kuntz^13^, Timothy Blake Palculict^14^, Safdar Abbas^15^, Muhammad Umair^16,17^, Mohammed AlMuhaizea^18^, Dilek Colak^19^, Hanan AlQudairy^20^, Maysoon Alsagob^20,21^, Catarina Pereira^22^, Roberta Trunzo^22^, Vasiliki Karageorgou^22^, Aida M. Bertoli-Avella^22^, Peter Bauer^22^, Arjan Bouman^1^, Lies H. Hoefsloot^1,2^, Tjakko J. van Ham^1,2^, Mahmoud Issa^23^, Maha S. Zaki^23^, Joseph G. Gleeson^24^, Rob Willemsen^1^, Namik Kaya^20^, Stefan T. Arold^3,25^, Reza Maroofian^9^, Leslie E. Sanderson^1#^, and Tahsin Stefan Barakat^1,2, 26,27#^

^1^ Department of Clinical Genetics, Erasmus MC University Medical Center, Rotterdam, The Netherlands

^2^ Whole Genome Sequencing Implementation and Research Task Force, Department of Clinical Genetics, Erasmus MC University Medical Center, Rotterdam, The Netherlands

^3^ Bioscience Program, Biological and Environmental Science and Engineering Division, Computational Bioscience Research Center, King Abdullah University of Science and Technology (KAUST), Thuwal 23955-6900, Saudi Arabia

^4^ Department of Cell Biology, Erasmus MC University Medical Center, Rotterdam, The Netherlands

^5^ Department for histology and embryology, Faculty of Medicine, University of Novi Sad, Serbia

^6^ Erasmus MC University Medical Center, Department of Neurology, Rotterdam, The Netherlands

^7^ Erasmus MC University Medical Center, Department of Internal Medicine, Rotterdam, The Netherlands

^8^ Institute of Clinical Chemistry and Clinical Pharmacology, University Hospital Bonn, Bonn, Germany

^9^ UCL Queen Square Institute of Neurology, Department of Neuromuscular Disorders, London, United Kingdom

^10^ NIHR Biomedical Research Centre, Wellcome Centre for Human Genetics, University of Oxford, Oxford, United Kingdom

^11^ Manchester Centre for Genomic Medicine, St Mary’s Hospital, Manchester University Foundation NHS Trust, Health Innovation Manchester, Manchester, United Kingdom

^12^ Division of Evolution, Infection and Genomics, School of Biological Sciences, Faculty of Biology, Medicine and Health, The University of Manchester, M13 9PL, Manchester, United Kingdom

^13^ Ann & Robert H. Lurie Children’s Hospital of Chicago, Division of Neurology, Division of Genetics, Chicago, United States of America

^14^ GeneDx, Gaithersburg, MD, 20877, United States of America

^15^ Department of Biological Science, Dartmouth College, Hanover, NH, United States of America

^16^ Medical Genomics Research Department, King Abdullah International Medical Research Center (KAIMRC), King Saud bin Abdulaziz University for Health Sciences, Ministry of National Guard Health Affairs, Riyadh, Saudi Arabia

^17^ Department of Life Sciences, School of Science, University of Management and Technology (UMT), Lahore, Pakistan

^18^ Neuroscience Centre, MBC: 76, King Faisal Specialist Hospital and Research Centre (KFSHRC), Riyadh, 11211, Saudi Arabia

^19^ Molecular Oncology Department, King Faisal Specialist Hospital and Research Centre (KFSHRC), Riyadh, 11211, Saudi Arabia

^20^ Translational Genomics Department, Center for Genomics Medicine, MBC: 26, PO Box: 3354, King Faisal Specialist Hospital and Research Centre (KFSHRC), Riyadh 11211, Saudi Arabia

^21^Applied Genomics Technologies Institute, King Abdulaziz City for Science and Technology (KACST), Riyadh, Saudi Arabia

^22^ CENTOGENE, GmbH, 18055 Rostock, Germany

^23^ Clinical Genetics Department, Human Genetics and Genome Research Institute, National Research Centre, Cairo, Egypt

^24^ Howard Hughes Medical Institute, University of California, Departments of Neurosciences and Pediatrics, Rady Children's Institute for Genomic Medicine, San Diego, United States of America

^25^ Centre de Biologie Structurale, CNRS, INSERM, Université de Montpellier, 34090 Montpellier, France

^26^ ENCORE Expertise Center for Neurodevelopmental Disorders, Erasmus MC University Medical Center, Rotterdam, The Netherlands

^27^ Discovery Unit, Department of Clinical Genetics, Erasmus MC University Medical Center, Rotterdam, The Netherlands

*equal contribution

^#^corresponding authors

Lead contact: [t.barakat@erasmusmc.nl](mailto:t.barakat@erasmusmc.nl)

# Author contributions

RD performed genomics and bioinformatics analysis, with help of SY and GG. EMS performed, and LES supervised zebrafish disease modeling, with help of NAK, HCvsL, SLZ, and TJvH. AN performed molecular biology experiments, with help of KL, YS and EMS. RR and STA performed structural protein modeling. MTM, GJGR, DL and EHJ performed metabolic investigations. IC provided human fetal samples and performed immunohistochemistry. RW performed electron microscopy analysis.

Patient recruitment and diagnosis was performed in the different families as follows: Family 1: AB, TSB and EMvdH phenotyped individuals 1 and 2, MvS, RvM, TvH and LHH performed diagnostic testing. Family 2 and 8: MI, MSZ phenotyped individuals 3, 4, 5, 19 and 20; HH, RM and JGG performed genetic investigations. Family 3: MoA, DC, HA, MaA and NKa performed genetic investigations and phenotyping of individuals 9, 10 and 11. FA performed phenotyping, and RT, VK and AMBA performed genetic investigations of individuals 6, 7 and 8. Family 4: AMBA, CP and PB performed genetic investigations and phenotyping of individual 12. Family 5: KM performed phenotyping of individuals 13, 14 and 15; AJ, ATP and SB performed analysis of WGS data generated within the Genomics England 100,000 Genomes project. Family 6: SA and MU phenotyped individual 16 and performed genetic investigations. Family 7: LDS, AS, NaK phenotyped individuals 17 and 18, TBP performed genetic diagnostics. TSB conceived and supervised the study. RD, EMS, LES and TSB wrote the manuscript, with input from all authors. All authors approved the final version of the manuscript.

# Supplementary Methods:

## **Patient Genetic investigations**

### Patient recruitment

All affected probands were investigated by their referring physicians and all genetic analyses were performed in a diagnostic setting. Affected probands or their legal guardians gave informed consent for genomic investigations and publication of their anonymized data including photographs and videos.

### Next-generation sequencing of index patients

**Family 1 (Individual 1-2)**: Genomic DNA was isolated from peripheral blood leukocytes of both affected siblings and used for whole genome sequencing using an Illumina Nova-seq 6000 platform (GenomeScan, Leiden, The Netherlands) with 150-bp paired-end reads. Whole genome sequencing reads were trimmed using Trim Galore! (version 0.6.6) (<https://www.bioinformatics.babraham.ac.uk/projects/trim_galore/>) to remove possible adaptor contamination and low quality reads. Trimed reads were aligned to GRCh37 using BWA (BWA-MEM version 0.7.17) [26]. Aligned reads were processed under the Genome Analysis Toolkit (GATK version 4.1.2.0) [33]. Best practice guidance and the GATK bundle were downloaded from ftp://[ftp.broadinstitute.org/bundle/hg38/](http://ftp.broadinstitute.org/bundle/hg38/). After removing duplicated reads and recalibrating the base quality scores, SNVs and indels were first called using GATK Haplotyper. Variant quality score recalibration and hard filtration were performed to generate the high-quality variant calls for SNVs and indels. The generated VCF files were further annotated using Variant Effect Predictor (VEP) [34]. Shared and non-shared ROH regions between affected and non-affected family members, respectively, were used to prioritize variants and were derived from SNP-array data obtained from routine clinical investigations. The identified AMFR variant was confirmed by Sanger sequencing, including segregation analysis in the family, and was also detectable by retrospective, manual assessment of the BAM files of the previously generated clinical exome and RNA-seq data, but at a low coverage. To calculate the median coverage over exons of *AMFR*, we used 100 randomly selected WES and 130 WGS samples from our in house database and used the “coverage” function of Bedtools (version 2.30.0) [39].

**Family 2 (Individual 3-5)**: Extensive genetic investigations in Family 2 included whole genome and exome sequencing from whole blood derived DNA and RNA-seq on fibroblasts in individual 3; whole genome sequencing and exome sequencing from whole blood derived DNA in individual 4; and whole genome and exome sequencing from blood derived DNA and RNA-seq on fibroblasts in individual 5. Both parents were subjected to whole genome sequencing from whole blood derived DNA. Analysis was performed as previously described [13], yielding no other likely disease explaining candidate than *AMFR*.

**Family 3 (Individual 6-11)**: Exome sequencing was performed in individuals 9, 10 and 11 as previously described [2], followed by routine segregation analysis by Sanger sequencing. Individuals 6, 7 and 8 and their family members were subjected to targeted Sanger sequencing of the familial *AMFR* variant performed at Centogene GmbH.

**Family 4 (Individual 12)**: Individual 12 was subjected to diagnostic solo whole exome sequencing analysis at Centogene, including CNV and Mitochondrial analysis. Exome sequencing was performed at Centogene GmbH as previously described [50]. Parental DNA was not available for segregation analysis.

**Family 5 (Individual 13-15)**: Family 5 participated in the Genomics England 100,000 Genomes project.[12] Libraries were prepared from blood DNA using the TruSeq PCR-Free High Throughput kit and sequencing was with 150bp paired-end reads in a single lane of an Illumina HiSeqX instrument. Reads were aligned to GRCh38 using the iSAAC Aligner (v03.16.02.19) [40].

**Family 6 (Individual 16)**: WES was performed using the Agilent SureSelect Target Enrichment Kit, and libraries were sequenced using an Illumina HiSeq 2500 platform (Illumina, San Diego, CA, US), followed by analysis as previously described [55]. Routine Sanger sequencing confirmed segregation of the variant in the family.

**Family 7 (Individual 17-18)**: Using genomic DNA from individual 17-18 and their parents, the exonic regions and flanking splice junctions of the genome were captured using the IDT xGen Exome Research Panel v1.0 (Integrated DNA Technologies, Coralville, IA). Massively parallel (NextGen) sequencing was done on an Illumina system with 100 bp or greater paired-end reads. Reads were aligned to human genome build GRCh37/UCSC hg19, and analyzed for sequence variants using a custom-developed analysis tool. The identified *AMFR* variant was confirmed by Sanger sequencing and segregation analysis. Additional sequencing technology and variant interpretation protocol have been previously described[41]. The general assertion criteria for variant classification are publicly available on the GeneDx ClinVar submission page ([http://www.ncbi.nlm.nih.gov/clinvar/submitters/26957/](https://eur01.safelinks.protection.outlook.com/?url=http%3A%2F%2Fwww.ncbi.nlm.nih.gov%2Fclinvar%2Fsubmitters%2F26957%2F&data=05%7C01%7Ct.barakat%40erasmusmc.nl%7C42ef3d4538474c08c9b008dad3ed7d45%7C526638ba6af34b0fa532a1a511f4ac80%7C0%7C0%7C638055314254141018%7CUnknown%7CTWFpbGZsb3d8eyJWIjoiMC4wLjAwMDAiLCJQIjoiV2luMzIiLCJBTiI6Ik1haWwiLCJXVCI6Mn0%3D%7C3000%7C%7C%7C&sdata=W4XUb84fOQ6T8a%2BCzdV25yqeL%2F63JN%2BX1JNa%2FC4PsVU%3D&reserved=0)).

**Family 8 (Individual 19-20)**: Blood-derived DNA of the two affected siblings was subjected to whole exome sequencing, routinely performed at the Broad Institute, as previously described [13].

## **Structural protein modeling**

We used VarSite [24], PDBsum [23], Uniprot [52] and AlphaFold [53] databases and PyMOL [1] to explore, analyze and study the variants at the protein structural level. The structure of the main isoform was taken from the AlphaFold database, entry ID Q9UKV5. To eliminate clashes of the non-membrane-bound domains with the membrane region, and for better visibility, we manually rearranged the region C-terminal to the RING domain in a stereochemically plausible way. The structure of the isoform uc002eix.3 was predicted using the AlphaFold2 v2.0. server implementation at KAUST [36]. The prediction was highly confident for the canonical domains, but of low confidence for the new N-terminal sequence (the average pLDDT score for uc002eix.3 was 60.7, and the pLDDT values for the new N-terminal sequence, the CUE, G2BR and VIM were 44.2, 88.0, 86.7, and 88.3, respectively).

## **Fibroblast cell culture**

Patient fibroblasts from Family 1 (individual 1, individual 2 and the unaffected healthy mother) were obtained using a punch biopsy according to standard procedures at the Erasmus MC, upon informed consent (IRB approval MEC-2017-341). Patient fibroblasts of Family 8 (individual 20 and the unaffected healthy mother) were obtained for diagnostic investigations in a clinical setting in Egypt, and upon consent by patients and their legal guardians the remaining material was re-purposed for research investigations. Patient fibroblasts were cultured in DMEM (1x) medium (Gibco) supplemented with 15% fetal calf serum, 100 U/ml penicillin and 100 µl/ml streptomycin, and MEM non-essential amino acids (Sigma) at standard conditions in cell culture incubators, as done previously [4]. For rescue experiments, a wild type AMFR expression plasmid (pcDNA-gp78-FLAG, Addgene #62370), or a plasmid expressing a RING mutant AMFR (pCDNA-gp78-FLAG RING mutant C356G H361A, Addgene #61751), both kind gifts from Yihong Ye, were used, and transfected into fibroblasts using Lipofectamine 3000.

## **Human fetal samples and immunohistochemistry**

Human fetal samples have been previously described [38]. Tissue was obtained, upon informed consent, and used in a manner compliant with the Declaration of Helsinki and the Research Code provided by the local ethical committees. Fetal brains were preserved after spontaneous or induced abortions with appropriate written consent for brain autopsy and use of rest material for research. We performed a careful histological and immunohistochemical analysis, and evaluation of clinical data (including genetic data, when available). We only included specimens displaying a normal cortical structure for the corresponding age and without any significant brain pathology. For immunohistochemical analysis, we cut 4 µm thin sections from formalin-fixed, paraffin-embedded whole fetuses (GW6 and GW9). Slides were stained with mouse anti-AMFR (HPA029018, Sigma; Germany) in a 1:50 dilution and visualized using the EnVision Detection SystemsPeroxidase/DAB, Rabbit/Mouse IHC kit (DAKO; Germany). Mayer’s hematoxylin was used as a counterstain for immunohistochemistry, followed by mounting and coverslipping (Bio-Optica; Italy) for slides. Prepared slides were analyzed and scanned under VisionTek® Live Digital Microscope (Sakura; Japan). Anatomical regions were determined according to the atlas of human brain development [6, 7].

## **Genome engineering of human embryonic stem cells**

H9 human embryonic stem cells (ESCs) were cultured, as previously described [5], in feeder-free conditions in mTeSR-1 medium (STEMCELL technologies) on Matrigel (Corning) coated plates. To generate *AMFR* knockout clones, we used our established CRISPR-Cas9 protocol [38, 44]. In short, H9 ESCs were transfected using Lipofectamine Stem Reagent (Invitrogen) with plasmids expressing two gRNAs targeting exon 4 (encoding AMFR main isoform residue 172-222), of *AMFR* (see **Supplementary Table 6** for oligonucleotide sequences) and either eSpCas9-t2A-GFP or eSpCas9-t2A-mCherry (kind gifts of Feng Zhang). 24h post-transfection, 5000 single GFP and mCherry double-positive cells were sorted and plated on a layer of Matrigel in a six-well plate in the presence of 10 µM ROCK-inhibitor (Millipore). After a week, single colonies were manually picked, expanded and genotyped using Sanger sequencing (see **Supplementary Table 6** for oligonucleotide sequences). All cell lines used in this report were regularly checked for the presence of mycoplasma and were negative during all experiments. Of note, multiple rounds of targeting exon 1, 2 and 7 did not result in knockout clones that had fully abolished AMFR expression, likely due to inefficient gRNAs and difficulties in designing optimal sequencing primers (data not shown).

## **Neural stem cell differentiation**

H9 human ESCs were differentiated into neural stem cells (NSCs) using a modified dual SMAD inhibition protocol as previously described [38]. Briefly, 18,000 cells/cm^2^ were plated on Matrigel-coated dishes in the presence of mTeSR-1 medium supplemented with 10 µM ROCK-inhibitor. The medium was changed daily until cells reached 90% confluency, then mTeSR-1 medium was switched to differentiation medium (KnockOut DMEM (Gibco), 15% KnockOut serum replacement (Gibco), 2mM L-glutamine (Gibco), MEM non-essential amino acids, 0.1 mM β-mercaptoethanol, 100 U/ml penicillin and 100 µg/ml streptomycin). Differentiation medium was supplemented with 2 µM A83-01 (Tocris) and 2 µM Dorsomorphin (Sigma-Aldrich) and was changed daily. At day 6, differentiation medium was changed to a 1:1 ratio of differentiation medium and NSC medium (KnockOut DMEM-F12 (Gibco), 2 mM L-glutamine, 20 ng/ml bFGF (Peprotech), 20 ng/ml EGF (Peprotech), 2% StemPro Neural supplement (Gibco), 100 U/ml penicillin and 100 µg/ml streptomycin), supplemented with 2 µM A83-01 and 2 µM Dorsomorphin. At day 10, cells were passaged using Accutase (Sigma) and were cultured in NSC medium. To assess the efficiency of NSC differentiation, qRT-PCR analysis for pluripotency markers and genes expressed in NSCs was performed (see **Supplementary Table 6** for primer sequences). As a control, commercially available H9-derived NSCs (Gibco) (a kind gift from Raymond Poot, Rotterdam) were used. For rescue experiments, the same wild type and RING mutant AMFR expression plasmids, as described above for fibroblast experiments were used. NSCs were transfected using Lipofectamine Stem Reagent following manufacturer’s instructions. 24 hour post-transfection, cells were harvested for RNA isolation and fixed with 4% PFA for lipid staining. For Tunicamycin treatment, NSCs at 80% confluence were cultured in duplicate in the presence of Tunicamycin (Sigma) dissolved in DMSO. Three concentrations were used (0 ng/µl; 2000 ng/µl; 4000 ng/µl), where each cell line received the same amount of vehicle DMSO. 24 hour post treatment cells were harvested for RNA and analyzed by qRT-PCR analysis.

## **qRT-PCR analysis**

Total RNA was isolated using TRI reagent (Sigma) and cDNA was synthesized using the iScript cDNA Synthesis kit (Bio-Rad). For RNA isolation from 5 dpf (days post fertilization) zebrafish larvae, samples were directly mixed with TRI reagent and homogenized using a disposable hypodermic needle (22 G). RNA was extracted separately from whole larvae (15 larvae per sample and 3 biological replicates per genotype) as well as isolated brains and the remaining bodies of >10 larvae per genotype (4 biological replicates for WT larvae and 5 biological replicates for *amfra-/-* larvae). qRT-PCR was performed using iTaq Universal SYBR Green Supermix (Bio-Rad) in a CFX96RTS thermal cycler (Bio-Rad). All oligonucleotides used are provided in **Supplementary Table 6**. Relative gene expression was determined following the ∆∆Ct method.

## **RNA-sequencing and data analysis**

For patient RNA-seq, fibroblasts obtained from Family 1 (index patient 1, 2 and his mother) were cultured in the presence or absence of cycloheximide, and RNA was isolated following standard diagnostic procedures at the diagnostics unit of the Erasmus MC Clinical Genetics department. RNA-seq and data analysis occurred in a diagnostic setting, and sequencing was performed at GenomeScan (Leiden, The Netherlands), as previously described [17]. For NSC RNA-seq, at passage 5, differentiated NSCs in six-wells from two wild type H9 derived cultures and 3 independent *AMFR* KO clones were collected and RNA was isolated using TRI reagent (Invitrogen) following manufacturer’s instructions. Obtained RNA was purified using column purification (Qiagen, #74204). mRNA capture, library prep including barcoding and sequencing on an Illumina HiSeq2500 machine were performed according to standard procedures at the Erasmus MC Biomics facility. Approximately 20 million reads were obtained per sample, with each biological replicate sequenced in two technical replicates.

FASTQ files obtained after de-multiplexing of single-end, 50-bp sequencing reads were trimmed using Trim Galore! (version 0.6.6) (<https://www.bioinformatics.babraham.ac.uk/projects/trim_galore/>) to remove possible adaptor contamination and low quality reads. Trimmed reads were aligned to the human GRCh38 reference genome using STAR (version 2.7.7) [16] and fragments per gene were counted using featureCounts (version 2.0.1) [28] from aligned reads. Reads Per Kilobase per Million mapped reads (RPKM) was calculated using edgeR after removing low-expressed genes and normalizing data [42]. To assess general NSC differentiation performance (**Supplemental Figure 6E**), we visually assessed RPKM expression levels of a panel of ESC and NSC specific genes, as done previously [38]. To generate these cell type specific gene lists, we previously retrieved genes annotated in the following GO terms using the GSEA/MSigDB web site v7.0: GO_FOREBRAIN_NEURON_DEVELOPMENT (GO:0021884), GO_CEREBRAL_CORTEX_DEVELOPMENT (GO:0021987), GO_NEURAL_TUBE_DEVELOPMENT (GO:0021915), BHATTACHARYA_EMBRYONIC_STEM_CELL [9] and BENPORATH_NOS_TARGETS [8]. To compare gene expression between groups, differential gene expression analysis for knockout and control NSCs was performed using edgeR [42]. The cut-off of significant differences in gene expression between groups was FDR < 0.05. Enrichr was used to assess functional enrichment of differentially expressed genes [21], used with default parameters and whole genome set as background. **Supplementary Table 4**, reports all outputs in p value, adjusted p value (q value) and combined score (which is the estimation of significance based on the combination of Fisher's exact test p value and z score deviation from the expected rank) for Enrichr. The top-2 significant enrichments of WikiPathyways_2021_human in the up- and down-regulated gene-sets were visualized using the GOChord function of the GOplot package (version 1.0.2) [54]. For Gene set enrichment analysis (GSEA), the WikiPathways subset of canonical pathways (CP) was downloaded from the Human MSigDB database (v2022.1, <http://www.gsea-msigdb.org/gsea/msigdb/download_file.jsp>). The matrix with gene symbols and the fold change of gene expression was used as the input of GSEA. The GSEA function of the clusterProfiler package was used to determine whether the predefined WikiPathways were significantly different between wild type and *AMFR* knockout NSC. Ridgeplot and gseaplot2 functions were used to visualize the GSEA results [29, 49, 57].

## **Western blotting**

Proteins of H9 ESC pellets were extracted using NE buffer (20 mM HEPES, pH=7.6, 1.5 mM MgCl_2_, 350 mM KCl, 0.2 mM EDTA and 20% glycerol), supplemented with 0.5% NP40, 0.5 mM DTT, cOmplete Protease Inhibitor Cocktail (PIC) (Roche) and 150 U/ml benzonase (Sigma). Pellets of fibroblasts were lysed using M4 buffer (50 mM HEPES, pH=7.4, 150 mM NaCl, 100 mM NaF, 0.5% Triton X-100, PIC). Protein concentration was determined by BCA (Pierce) and 30 µg (H9 ESC) or 50 µg (fibroblasts) of proteins were loaded into a 4-15% Criterion TGX gel (Bio-Rad). The TransBlot Turbo Transfer system (Bio-rad) was used to transfer proteins to a nitrocellulose membrane. The membrane was blocked with 5% milk in TBS and then incubated overnight at 4˚C with primary antibody diluted in 5% BSA in TBS containing 0.1% Tween (TBS-T). After a few TBS-T washes, the membrane was incubated at room temperature (RT) for 1 hour in secondary antibodies diluted in 5% milk in TBS-T. Images were obtained using an Odyssey CLX scanning system (Li-Cor) and band intensities were quantified using Image Studio (Li-Cor). Antibodies used were: Ms-α-Vinculin (sc-59803) 1:10,000; Rb-α-AMFR (Cell Signaling Technology, #9590) 1:1000 (H9 ESC) or 1:200 (fibroblasts), IRDye 800CW Goat anti-mouse (Li-Cor, 926-32210) 1:5000, IRDye 680 Goat anti-rabbit (Li-cor, 926-32221) 1:5000.

## **Oil Red O staining in human cells**

Oil Red O (ORO, Sigma) was kept as a stock solution of 0.6% (w/v) in 100% isopropanol. A 0.4% working solution was prepared by diluting 3 parts of ORO stock solution in 2 parts of dH_2_O, followed by incubation for 15 min at RT and filtering through a 0.2 µm filter attached to a syringe. Fibroblasts were cultured on a coverslip and maintained in fibroblast medium. Differentiated NSCs were cultured on coverslips pre-coated overnight with 100 µg/ml poly-d-lysine (Sigma) and further coated with Matrigel (Corning) for 1 hour at 37 °C, and maintained in NSC medium. At 60-80% confluency, cells were fixed with 4% PFA for 15 min at RT. Fixed cells were then stained with the ORO working solution for 20 min and counterstained with hematoxylin solution (Klinipath) for 5 min. Every staining step was followed by a 5 min wash with PBS. Coverslips were then mounted on object glasses using ProLong Gold antifade reagent (Invitrogen). Images were acquired with an Olympus BX40 microscope and image quantification analysis was done in RStudio and ImageJ (FIJI) [45].

## **Electron microscopy**

Each patient-derived fibroblast line was cultured in two T75 flasks in fibroblast medium. At 90% confluency cells were collected in a 15 ml falcon tube (a cell pellet of 1-1.5 cm, equaling approximately 12 million cells) and the supernatant was removed. Without disturbing the pellet, cells were then fixed with 1.2% glutaraldehyde – 4% formaldehyde for at least a week at 4°C. Subsequent processing for electron microscopy was performed using routine diagnostic procedures at the Pathology Department of the Erasmus MC. In short, samples were washed in 0.1 M sodium cacodylate buffer and postfixed in 2% (w/v) OsO_4_. Samples were dehydrated with a graded series of washes in acetone, transferred to acetone/Epon solutions, and eventually embedded in Epon. Ultra-thin sections (approximately 60 nm thin) obtained with a Reichert-Jung Ultracut E microtome were collected on copper slot grids. Sections were contrasted with uranyl acetate and lead citrate and examined with a Philips CM10 transmission electron microscope, operated at an accelerating voltage of 80 kV. Each fibroblast line was analyzed in two technical replicates. Fibroblasts of a healthy individual were used as a control. For zebrafish experiments, 5 dpf larvae were fixed with 1.2% glutaraldehyde – 4% formaldehyde for at least a week at 4°C before being processed using the same routine diagnostic procedures at the Pathology Department of the Erasmus MC as described above. The brains of two *amfra*-/- larvae and three wildtype control larvae were imaged from sagittal sections.

## **Immunocytochemistry in human cells**

Fibroblasts were seeded on coverslips, cultured at 80% confluence and fixed with 4% PFA for 15 min. Coverslips were then incubated for 15 min at RT in permeabilization solution (0.5% Triton in PBS), followed by incubation in blocking solution (3% BSA in PBS) for 1 hour. Coverslips were incubated with an Ms-ɑ-LAMP2 antibody (DSHB, H4B4, 26 µg/ml - a kind gift of Gerben Schaaf, Erasmus MC) diluted 1:13 in blocking solution, overnight at 4°C. The next day, coverslips were washed thrice with PBS and incubated using the secondary Cy3-donkey anti-mouse IgG antibody (Jackson, 715-165-150), diluted at 1:200 in blocking solution, for 1 hour in the dark. ProLong Gold antifade reagent with DAPI (Invitrogen) was used to mount the coverslips on object glasses. Images were acquired using a ZEISS Axio Imager M2 microscope with 63X objective.

## **Zebrafish husbandry**

For all experiments only zebrafish larvae <120 hours post fertilization (hps) were used. No animal experimentation occurred with adult zebrafish. Adult zebrafish were only used for routine breedings and handling procedures at the Erasmus MC animal facility as approved by the Animal Experimentation Committee at Erasmus MC, Rotterdam.

Adult breeding stocks were maintained at 28°C on a 14/10h light/dark cycle following standard husbandry guidelines. Zebrafish embryos and larvae were kept at 28°C on a 14/10h light/dark cycle in 1 M HEPES buffered (pH 7.2) E3 medium (34.8 g NaCl, 1.6 g KCl, 5.8 g CaCl_2_ · 2H_2_O, 9.78 g MgCl_2_·6 H_2_O). In order to prevent pigmentation in embryos used for immunohistochemistry or Oil Red O staining, the medium was changed at 1 dpf to fresh E3 + 0.003% 1‐phenyl 2‐thiourea (PTU). For all other experiments, media was refreshed at 1 dpf with E3.

For statin treatment, 20 embryos at 8 hpf were placed per group in 6-well plates filled with 4 ml of media. The stock solution of 20 mg/ml simvastatin (SMV, Cayman Chemicals 10010344) was prepared in 100% EtOH and the stock solution of 15 mg/ml atorvastatin (ATV, BioVision 2278-10) was prepared in 100% DMSO. Each was then diluted in E3 medium to a final working concentration of 0.3 nM SMV (0.0063% final concentration of ethanol) or 0.3 µM ATV (0.11% final concentration of DMSO). Statin concentrations were chosen following extensive titrations (data available upon request), selecting the highest dose that did not visibly impact morphology or survival by 5 dpf, and control solutions were included using the final working concentration of each vehicle. All media was refreshed at 3 dpf.

## **CRISPR-Cas9 Genome editing in zebrafish**

In order to disrupt *amfra*, exon 1 was targeted using a Cas9/gRNA RNP complex using published protocols [20]. In brief, fertilized oocytes from a wild type AB control strain were injected at the single-cell stage with Cas9 protein complexed with crRNA:tracrRNA (Integrated DNA Technologies, Alt-R-CRISPR-Cas9 System)*.* These putative P0 larvae were grown to adulthood and screened for germline transmission of variant alleles by Sanger sequencing of individual embryos obtained from intercrosses. This identified a founder carrying a 5 bp deletion allele, subsequently named *amfra^re31^*. Adults from successive generations were genotyped by Sanger sequencing of gDNA extracted from fin clips until homozygosity was reached. For this study, experiments were performed on embryos and larvae generated from incrosses of *amfra^re31/re31^* adults, referred to for simplicity as *amfra-/-*.

## **Oil Red O staining in zebrafish**

To prepare the ORO working solution, the same ORO stock solution used for cells was mixed 1:1 with 10% isopropanol. The solution was mixed by rotation at room temperature for 15 minutes and filtered before use. Whole larvae were fixed at 3 dpf with 4% paraformaldehyde at 4°C overnight. The fixed 3 dpf larvae were washed first quickly with PBS followed by an incubation in 60% isopropanol for 1 hour, after which this was replaced with 1 mL of ORO working solution for 1 hour at room temperature. After discarding the solution, the larvae were quickly washed with 60% isopropanol and rinsed again for 3 min in 60% isopropanol, followed by a quick wash in 0.1% PBS-T (PBS with 0.1% Tween 20). The stained larvae were subsequently washed with increasing concentrations of glycerol (30%, 50%, and 75% diluted in PBS-T, each for 15 min at room temperature), prior to storage in 100% glycerol at 4°C. Brightfield images were taken with an Olympus SZX16 Microscope and processed using FIJI. For the quantification of the ORO staining, FIJI software was used [45] and regions of interest (ROIs) were selected for analysis as indicated. The images were processed using the color deconvolution2 plugin [22, 43] with the RGB vector and 32-bit absorbance output. The average optical integrated density (OID) across each ROI was quantified using the red channel, which reflects the color spectrum of the ORO staining. An average background signal for each image was determined from randomly selected ROIs that did not overlap with the imaged embryo, which was then subtracted from the ORO OID measurement.

## **Immunohistochemistry on zebrafish embryos**

For zebrafish experiments, 2 dpf whole embryos were fixed with 4% paraformaldehyde at 4°C overnight. Embryos were washed twice with PBS-T (2% triton) for 5 min and dehydrated in 70% ETOH at 4% overnight. Samples were rehydrated with PBS-T for 5 min and blocked with 1% BSA 0.1% DMSO in PBS-T at room temperature for 2 hours. Embryos were incubated overnight at 4°C with the primary antibody (mouse anti-acetylated tubulin, Sigma T6793) diluted 1:500 in 5% BSA in PBS-T. Samples were quickly rinsed twice and then washed 10 x 30 min with PBS-T before being incubated overnight at 4°C in secondary antibody (Alexa 488 anti-mouse, Jackson 715-547-003) diluted 1:200 in 2% BSA in PBS-T. Finally, embryos were washed with PBS-T (6 x 10 min) and mounted in low melting agarose to image with a Leica SP5 intravital imaging setup with a 20 × /1.0 NA water-dipping lens. Confocal z-series images were acquired and analyzed using the SNT plugin [32] (simple neurite tracer) in FIJI [45]. Neurons were traced and quantified as previously described [3].

## **Behavioral analysis**

Touch-evoked escape responses were induced by touching 3 dpf larvae with a hypodermic needle (22G) at the trunk. Behaviors were recorded using an Olympus SZX16 Microscope. Larvae were recorded individually in the middle of a petri dish containing E3 media. Swimming away from the needle and out of the observed area within 0.1 sec was considered the normal touch-evoked escape response [10, 11]. Larvae not moving out of the observed area in less than 0.1 sec were considered to have a delayed response, and no response was defined as larvae not reacting after being touched by the needle. Three experimental replicates were performed, each with 15 larvae per genotype and treatment group.

## **Sterol analysis**

Wild type and *amfra-/-* larvae at 5 dpf, grown in E3 medium or treated from 8 hpf onwards with simvastatin (SMV) or atorvastatin (ATV) or their respective vehicle controls ethanol (ETOH) or DMSO, were used to determine the levels of cholesterol and non-cholesterol sterols. For this analysis, either whole larvae or dissected brains and their remaining bodies were used. To obtain sufficient material for the analysis, each measured sample consisted of pools of 7-15 larvae (see **Supplementary Table 5** for the exact numbers per sample), and obtained values were normalized for the number of larvae per pool. Most samples of whole larvae were measured in independent biological replicates, whereas dissected brain and body samples were measured as single pools. Cholesterol was determined by gas-chromatography-flame ionization detection and non-cholesterol content was determined by gas chromatography-mass spectrometry-selected ion monitoring (GC-MS-SIM) as previously described[46, 48]. Briefly, tissues were dried in a speedvac concentrator (12 mbar; Savant AES 1000) and weighed. Cholesterol, cholesterol precursors and plant sterols were extracted using chloroform. After alkaline hydrolysis, the concentrations of the cholesterol precursors lanosterol, 24,25-dihydrolanosterol, lathosterol, and desmosterol and the plant sterols campesterol and sitosterol were measured with GC-MS-SIM in selected ion monitoring mode. The trimethylsilyl-ethers of the sterols were separated on a DB-XLB (30 m length x 0.25 mm internal diameter, 0.25 µm film) column (Agilent Technologies) using the 6890N Network GC system (Agilent Technologies). Epicoprostanol (Steraloids, Newport, RI, USA) was used as an internal standard, to quantify the non-cholesterol sterols (Medical Isotopes, Pelham, NH, USA) on a 5973 Network MSD (Agilent Technologies). Total cholesterol was measured by GC-flame ionization detection on an HP 6890 GC system (Hewlett Packard, Waldbronn, Germany), equipped with a DB-XLB (30 m length x 0.25 mm internal diameter, 0.25 µm film) column (Agilent Technologies) using 5a-cholestane (Steraloids) as internal standard.

## **Statistical analysis and data presentation**

For image processing and quantification, RStudio and FIJI [45] were used. Lipid droplets from the ORO stainings and the length of zebrafish larvae were manually quantified. The statistical test used to determine statistical significance is indicated in each figure legend. p<0.05 was considered significant. Asterisks indicate levels of significance (* = p<0.05; ** = p<0.01; *** = p<0.001; ns = not significant).

## **Data Availability**

RNA-Seq of NSCs is publicly available through the National Center for Biotechnology Information (NCBI) Gene Expression Omnibus (GEO) under accession number GSE202141. Genome sequencing data for family 5 is available in the National Genomic Research Library from the 100,000 Genomes Project for which researchers can apply for access at Genomics England. For the other families, due to privacy regulations and given consent under which patients were recruited, raw patient RNA-seq data and genomic sequencing data cannot be made available. Codes used for data analysis are available via GitHub: <https://github.com/barakatlab/AMFR_paper.git>

# Supplementary Notes

## **Possible ERAD independent pathomechanisms of AMFR dysfunction**

Interestingly, whereas overexpression of exogenous wild type AMFR in patient-derived fibroblasts and *AMFR* KO NSCs could fully rescue the lipid droplet size to a level similar to that in wild type cells, a RING domain mutant, expected to have lost E3 ubiquitin ligase activity [31], could still partially rescue. It thus remains possible that some of the pathomechanisms are mediated by ERAD independent roles of AMFR, although we cannot exclude that exogenous overexpression of the RING domain mutant AMFR still results in residual weak E3 ligase activity sufficient for partial rescue. Alternatively, as the p.Cys356Gly and p.His361Ala RING domain mutations do not affect other AMFR domains, the exogenous mutant AMFR protein is still able to recruit p97 through the VIM domain and through its TMD can bind to INSIG-1, required for sterol regulated ubiquitination of HMGCR [15, 27, 47]. In the absence of ERAD competent AMFR, INSIG-1 is stabilized [25]. Since the RING domain mutant AMFR can still bind INSIG-1, this might boost an alternative non-conventional pathway mediating E3 ligase activity that might result in partial lipid droplet phenotype rescue, which will not occur if no AMFR is expressed as in the background of patient-derived cells. Attractive candidates for this alternative source of E3 ligase activity are TRC8, RNF145 and HDR1 (alias SYVN1), which have also been described to play redundant roles in sterol-regulated degradation or basal turnover of HMGCR [18, 19, 35], although the exact contribution of each protein in these processes remains debated and might vary per cell type and species [51]. Future studies are required to decipher these mechanisms.

## **Differences in findings from *Amfr* knockout mouse models**

A Cre recombinase mediated, liver-specific knockout of floxed *Amfr* exons 5-8 on a C57BL/6J background led to increased levels of both HMGCR and Insig-1/-2, together resulting in a net decrease of lipid biosynthesis and protecting these mice from diet-/age-induced obesity and glucose intolerance [30]. A whole-body knockout, mediated by intercrossing of a CMV-promoter driven Cre recombinase strain with the same floxed allele, resulted in viable homozygous *Amfr-/-* offspring that appeared normal, although no detailed phenotypic characterization of these mice was reported [59]. Whereas Insig-2 was stabilized in the absence of Amfr in the liver of these animals resulting in downregulation of SREBP target genes, expression of Insig-2 protein levels was unaltered in muscle, indicating tissue specific differences in susceptibility of Amfr targets for ubiquitylation. Surprisingly, a whole-body knockout *Amfr* mouse on a C57BL/6 background created by insertion of a gene-trapping vector in *Amfr* intron 1, did not show significant upregulation of HMGCR or suppression of SREBP-1 in knockout hepatocytes. However, it showed upregulation of Insig-2 and increased susceptibility to ER-stress through SREBP-1 activation, causing obesity and ER stress-mediated hepatic steatosis in one year old mice with increased LDs, arguing that Amfr plays a critical role in ER stress-mediated ERAD [58]. Although a detailed phenotypic characterization was also not reported for this model, it yielded viable homozygous knockout offspring at Mendelian ratios[58], in contrast to other mouse models where eliminating critical components of the ERAD machinery led to lethality [37, 56].

# Supplementary Figures and Legends:


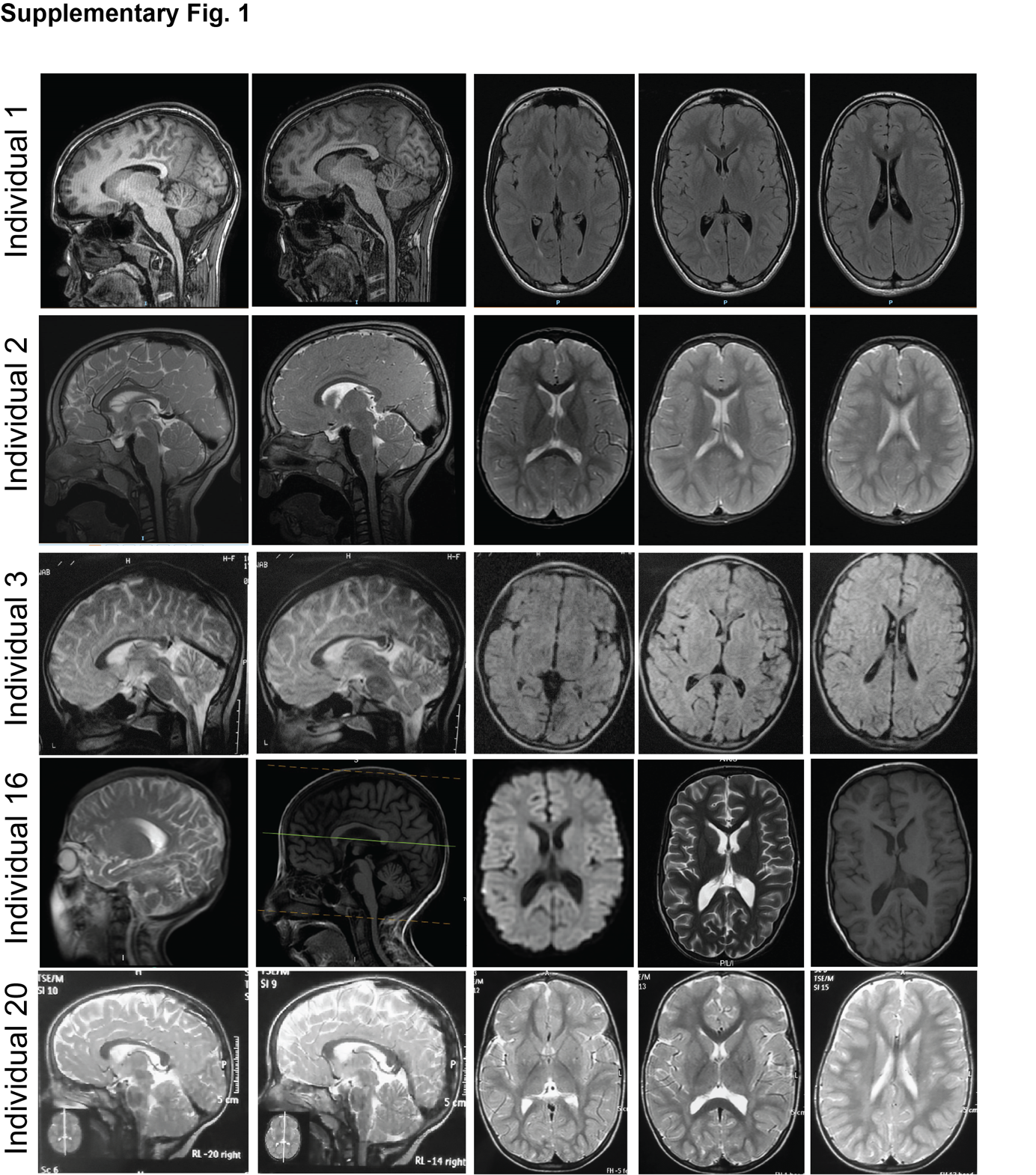


## **Supplementary Fig. 1**: MRI brain imaging in individuals with *AMFR* loss of function variants.

Representative axial and sagittal T1 or T2 weighted brain MRI images from individuals 1, 2, 3, 16 and 20. Individual 1 shows a T2 hyperintense lesion in the globus pallidus with an otherwise unremarkable brain morphology. No abnormalities are observed in individual 2. Individual 3 and 20 show thinning of the corpus callosum with otherwise unremarkable brain structure. In individual 16, T2/FLAIR hyperintensities are seen in the peripheral, subcortical areas with otherwise unremarkable brain structure.

## **
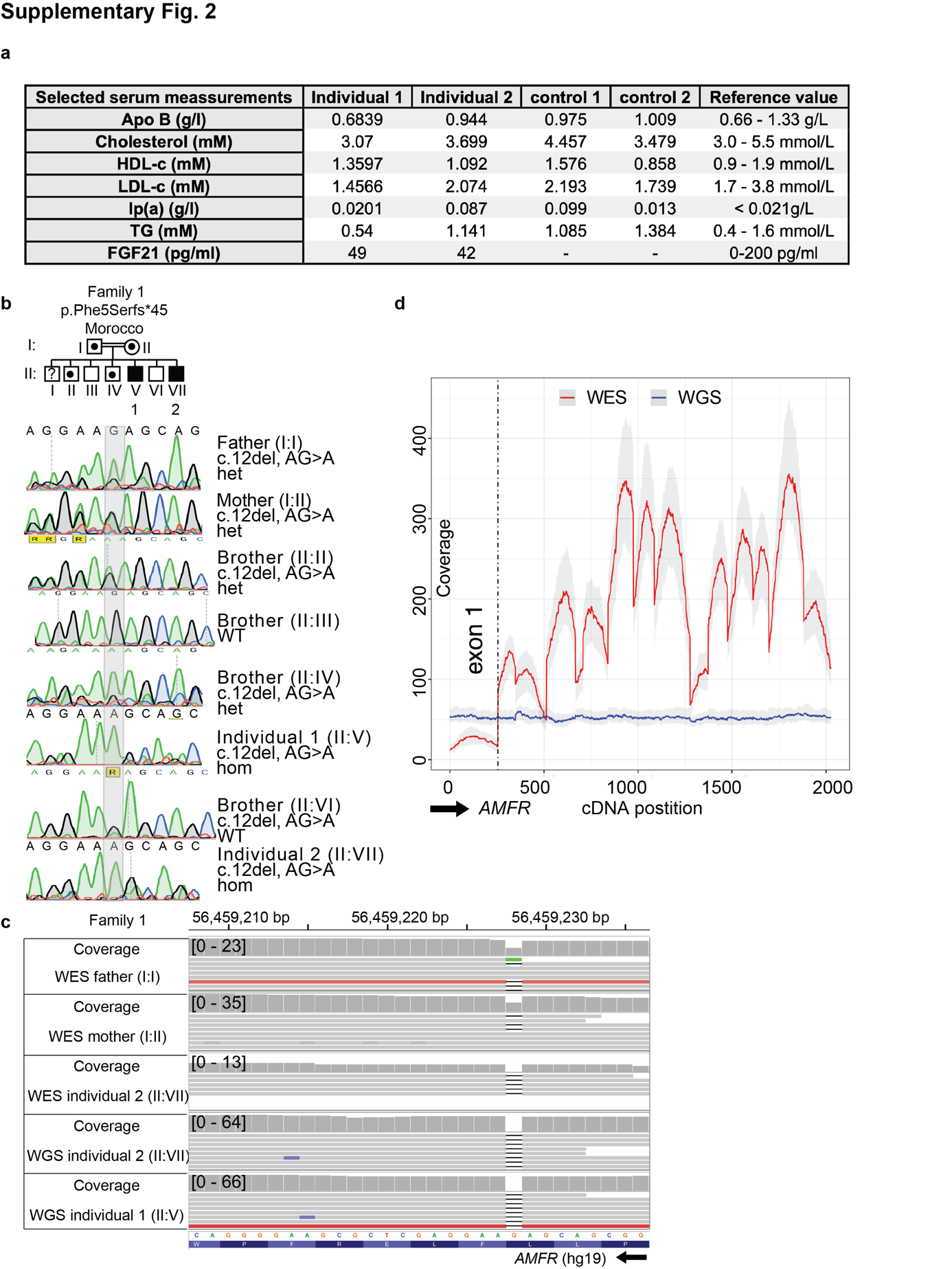
**

## **Supplementary Fig. 2**: Metabolic and genetic investigations in Family 1.

1. Metabolic measurements in plasma of individuals 1 and 2, and two unrelated controls. Apo B: apolipoprotein B; HDL-c: high-density lipoprotein cholesterol; LDL-c: low-density lipoprotein cholesterol; lp(a): A lipoprotein (a), TG: triglycerides; FGF21: Fibroblast growth factor 21.
2. Sanger sequencing chromatograms of Family 1, showing full segregation of the chr16(GRCh37):g.56459228del variant with the HSP phenotype, with only individual 1 and individual 2 homozygous for this one base pair deletion.
3. IGV browser view showing comparison of aligned WGS reads over the mutation site found in family 1, to the aligned reads of the previously performed trio WES analysis in individual 2. The homozygous chr16(GRCh37):g.56459228del variant was only present in 5 reads in the WES data, which is below the clinically used detection threshold of 10 reads to call a variant which is used in the clinical analysis pipeline.
4. Comparison of sequence coverage from 100 in house WES samples (red) and 130 in house WGS samples (blue) at the *AMFR* locus. The first exon of *AMFR* shows a poor sequence coverage in WES compared to WGS. The dashed vertical line indicates the end of the first exon of *AMFR*. Red or blue solid line is the median coverage, grey shading represents the interquartile range from 25th to 75th percentile.

**
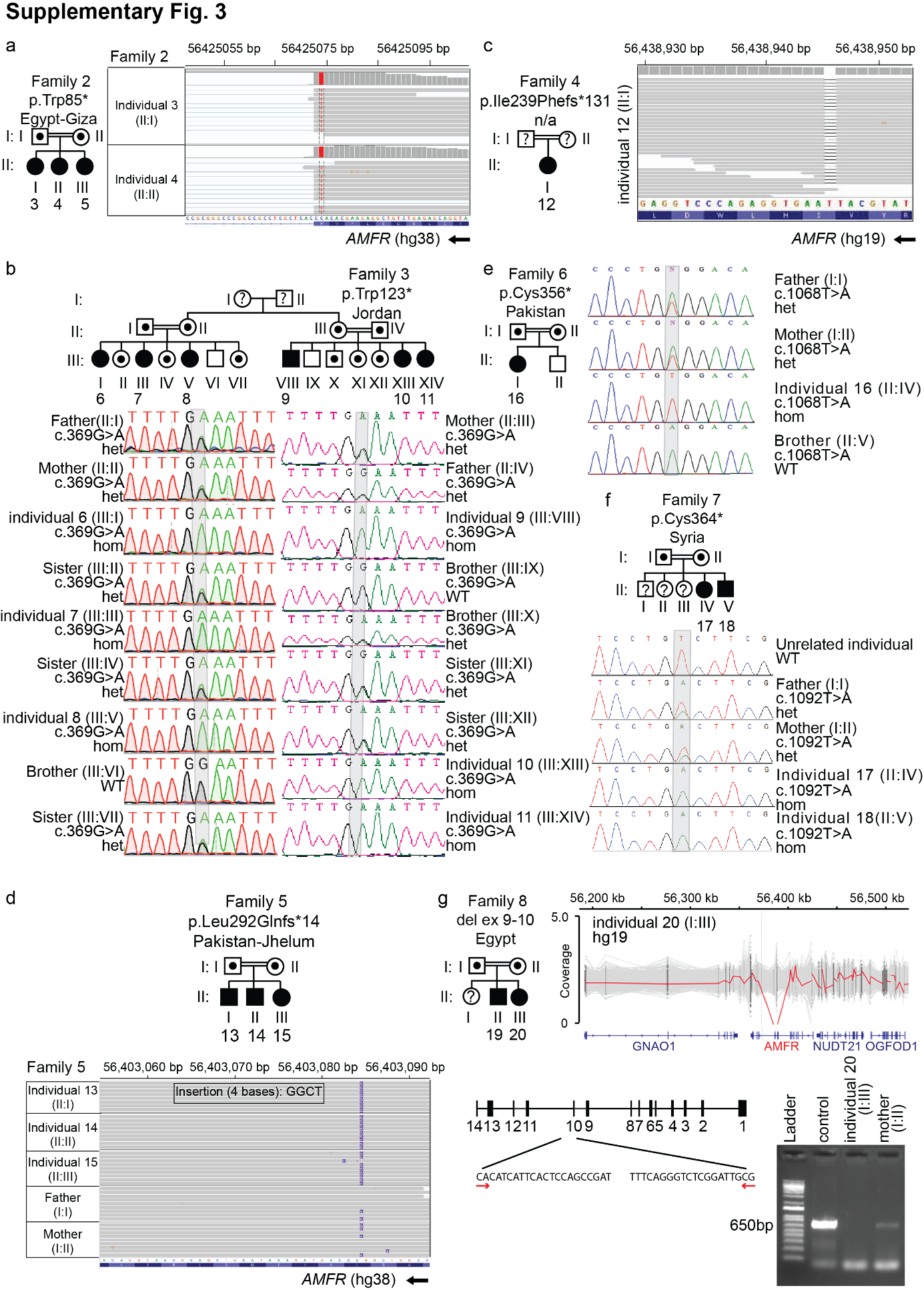
**

## **Supplementary Fig. 3:** Genetic investigations in families 2-8

1. IGV genome browser screenshot showing the p.Trp85* *AMFR* variant in individual 3 and 4 from Family 2. Sanger sequencing confirmed full segregation of the variant in the family (not shown). Similar to the variant in Family 1, the p.Trp85* *AMFR* variant was only reliable detected in WGS but missed in previous WES, likely due to the poor WES coverage of *AMFR* exon 1.
2. Sanger sequencing chromatograms of Family 3, confirming full segregation of the p.Trp123* *AMFR* variant in the family.
3. IGV genome browser screenshot showing the p.Ile239Phefs*131 *AMFR* variant in individual 12 from Family 4. Parental DNA was not available for segregation analysis.
4. IGV genome browser tracks of aligned WGS reads in Family 5, showing a 4 bp insertion in *AMFR* causing a frameshift (p.Leu292Glnfs*14), which is homozygous in the affected children and heterozygous in the healthy parents.
5. Sanger sequencing chromatograms of Family 6, confirming full segregation of the p.Cys356Ter *AMFR* variant in the family.
6. Sanger sequencing chromatograms of Family 7, confirming segregation of the p.Cys364* *AMFR* variant in the family. Only the affected individuals and their parents could be consented for genotyping.
7. WES analysis in Family 8, visualizing copy number calling from whole exome sequencing in the index individual 20 in the IGV genome browser, identifies the exon 9 to exon 10 deletion in *AMFR*. Note the specific drop in copy number in the exon 9 to exon 10 region of AMFR (hg38:chr16:56385545-56389473). Individual 19 showed similar results (not shown). Also shown is validation by PCR analysis, confirming the exon 9 to exon 10 deletion of *AMFR* in fibroblast-derived DNA from Family 8. Scheme shows the location of the used primers for genotyping.

**
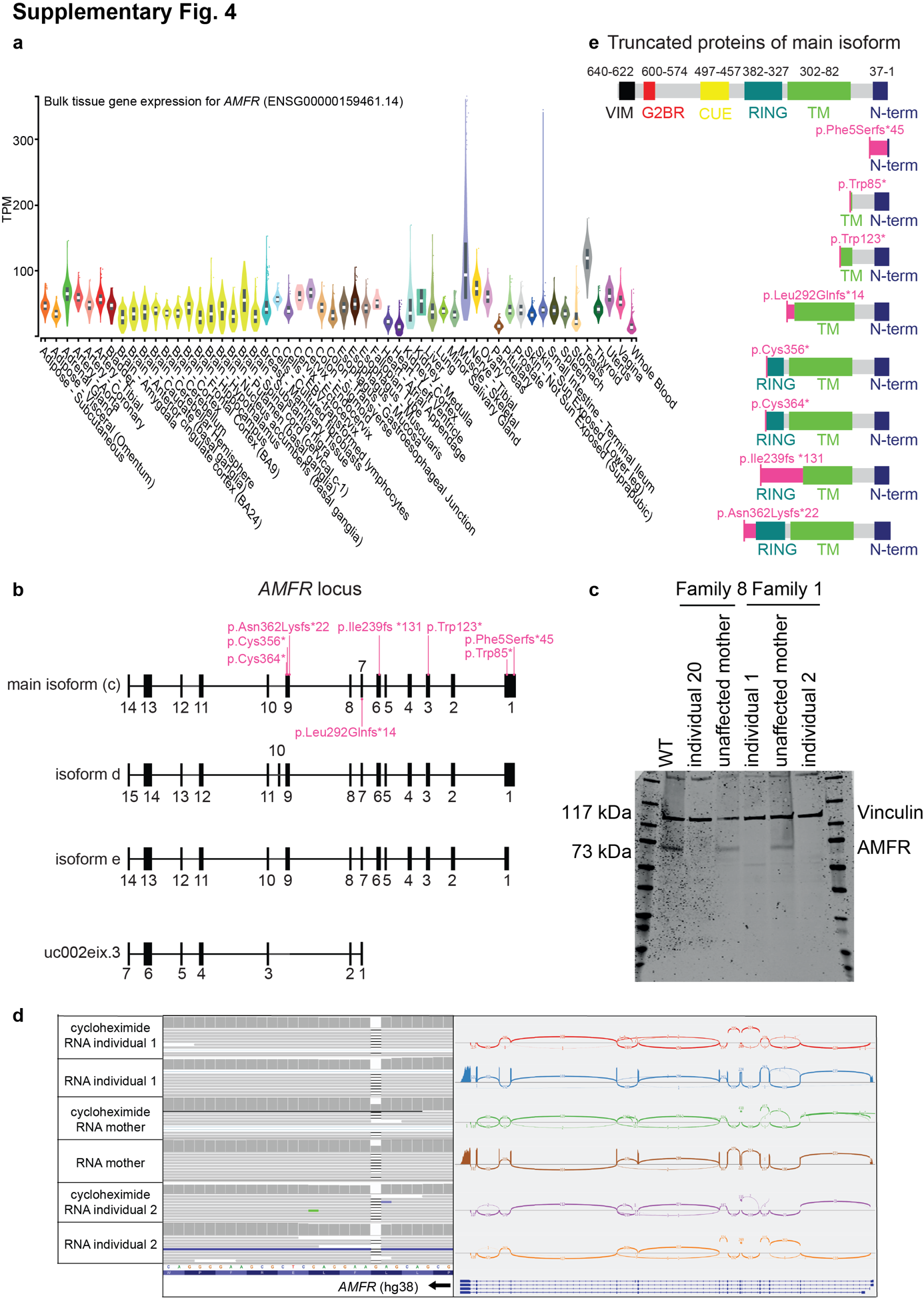
**

## **Supplementary Fig. 4:** *AMFR* is widely expressed and encodes potentially multiple isoforms of which the main isoform is predominant.

1. Violin plots showing distribution of *AMFR* gene expression (in TPM) amongst samples from the GTEx portal [14] for tissues and cell lines. Samples are sorted in alphabetical order. Outliers are indicated by dots.
2. Schematic drawing of the coding sequence of *AMFR* transcript isoforms in humans: isoform c (referred to as main isoform, NM_001144.6), isoform d (NM_001323512.2), isoform e (NM_001323511.2) and the truncated isoform uc002eix.3. The coding exons (numbered) are indicated as filled boxes. Also indicated are the mutation sites of the variants encountered in the *AMFR* patient cohort, indicated with the main isoform as reference. For a discussion of the structure of the proteins derived from the main transcript isoform and isoform uc002eix.3, see the main text and **Figure 1E**. Transcript NM_001323512.2 (isoform d) has an additional exon (between exon 9 and exon 10 of the main transcript (NM_001144.6, isoform c), leading to an insertion of a 32 amino acid peptide (DDSSMRVTAPEDVPVGQEVEVVDSDTDAPDLW) between Gly426 and Ser427 of the main transcript (NM_001144.6, isoform c), resulting in an otherwise identical 675 amino acid protein (76 kDa). This disordered peptide addition in which no clear ligand binding motif was found by ELM, is located between the RING domain (residues 327–382) and the CUE domain (residues 457–497) and might function as a spacer increasing accessibility of C-terminal AMFR domains when membranes have charged lipid headgroups. Transcript NM_001323511.2 (isoform e) results in a 548 amino acid protein of predicted 63 kDa that lacks the first 95 aa compared to the main transcript (NM_001144.6, isoform c). This causes the lack of the N-terminal signal sequence and the beginning of the TMD, which probably leads to an unstable or mislocalized protein.
3. Uncropped full length image of the Western blot shown in **Figure 1F**, detecting Vinculin (upper band, 117 kDa) and AMFR (lower band, 73 kDa) in patient-derived fibroblasts from family 1 and family 8, their healthy heterozygous mothers and an unrelated wild type control. Only expression of the main AMFR isoform at 73 kDa was reliably detected in controls. No expression of the main AMFR isoform is detected in patient samples, and no reliable expression of any possible truncated protein versions was noticed.
4. *AMFR* read coverage from clinical RNA-seq performed on patient-derived fibroblasts, cultured in the presence or absence of cycloheximide. Left panel shows aligned RNA-seq reads over part of *AMFR* exon 1 displayed in the IGV genome browser, from affected individual 1 and 2 (homozygous for the p.Phe5Serfs*45 variant) and the unaffected mother (heterozygous for the p.Phe5Serfs*45 variant). Right panel shows a sashimi plot over the whole *AMFR* locus. No difference between cycloheximide treated and untreated samples were observed. In particular, no difference in read number of the wild type and mutant allele was observed in the sample from the heterozygous mother, arguing against NMD.
5. Schematic drawing of the hypothetical truncated proteins derived from the main isoform that would result from the variants encountered in the *AMFR* patient cohort if truncated proteins would be stably expressed. Protein domains are colored corresponding to **Figure 1D**. Pink domains indicate the newly formed amino acids caused by the encountered variants.

## **
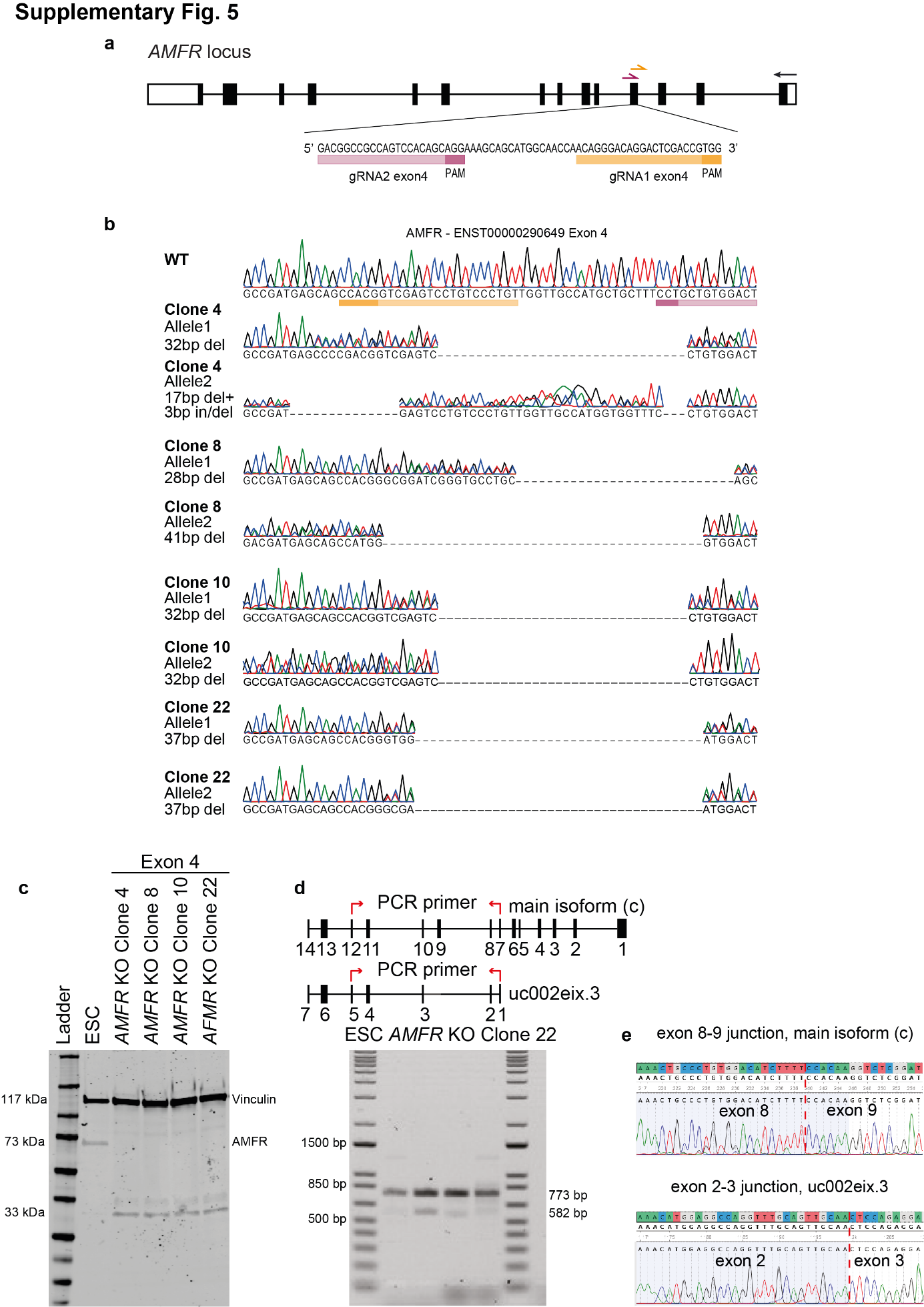
**

##

## **Supplementary Fig. 5:** Generation of *AMFR* knockout embryonic stem cells

1. Schematic drawing of the *AMFR* gene at chromosome 16q12.2, with coding exons indicated as filled boxes. Zoom-in shows target sequence and gRNAs targeting exon 4 that were used to generate *AMFR* KO ESCs.
2. Sanger sequencing chromatograms of wild type control ESCs and *AMFR* KO ESCs, confirming compound heterozygous indels in clones 4, 8, 10 and 22.
3. Uncropped full length image of the Western blot shown in **Figure 2B**, detecting Vinculin (upper band, 117 kDa) and AMFR (lower band, 73 kDa). L, ladder.
4. RT-PCR using primers indicated in the scheme, detecting expression of the main isoform (upper band, 773 bp) and the truncated isoform uc002eix.3 (lower band, 582 bp) in ESCs (example from *AMFR* KO clone 22, 4 RT-PCR replicates). Difference between band size is caused by the exclusion of main isoform exon 9 in the coding sequence of the truncated isoform uc002eix.3.
5. Sequencing chromatograms of RT-PCR products from panel D, confirming the main isoform c exon 8 to exon 9 junction present in the 772 bp product (upper panel), and the uc002eix.3 exon 2 to exon 3 junction present in the 582 bp product (lower panel).

**
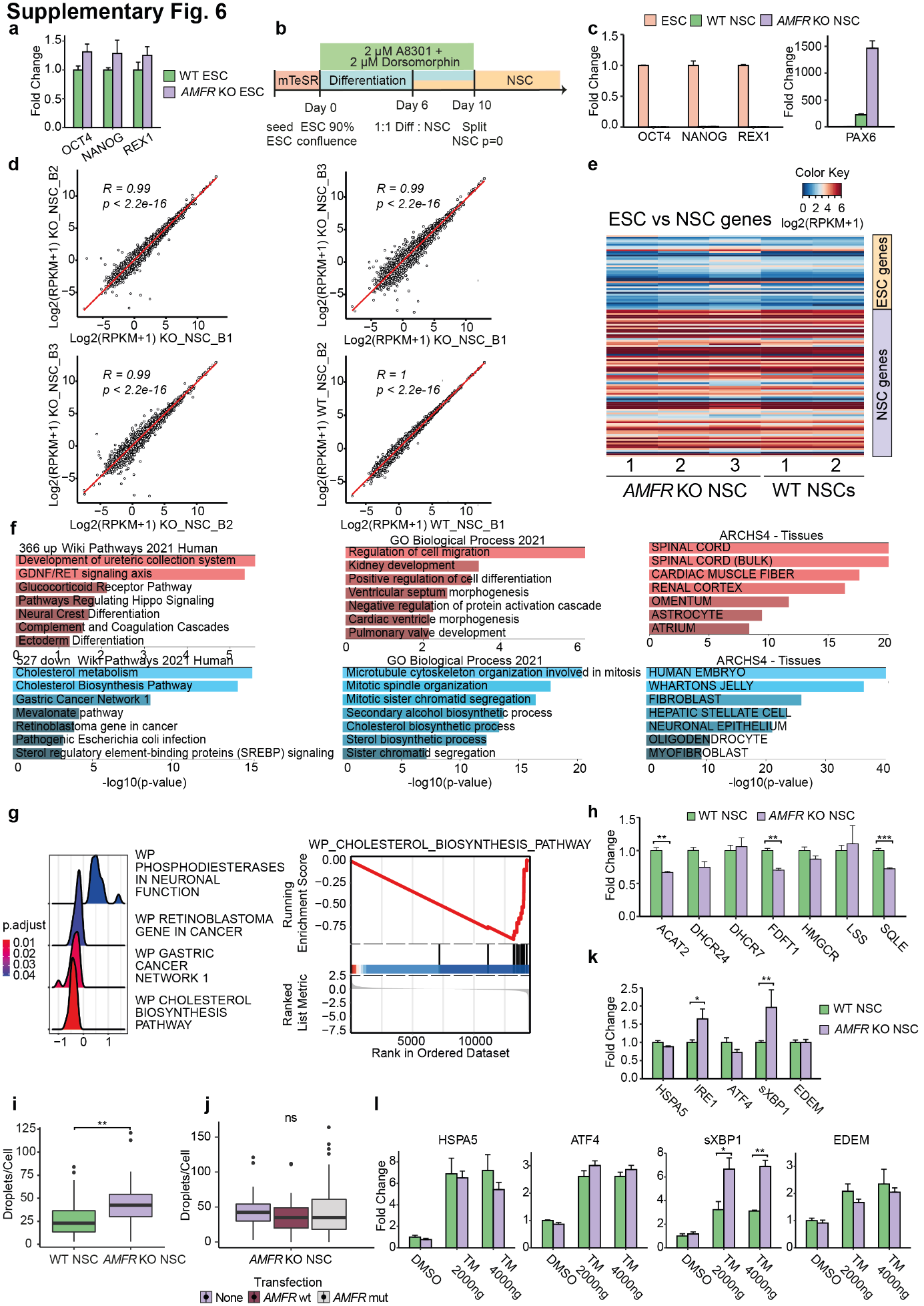
**

## **Supplementary Fig. 6**: *AMFR* knockout neural stem cells show alterations of cholesterol homeostasis.

1. qRT-PCR of pluripotency markers *OCT4, NANOG* and *REX1* of WT ESCs and *AMFR* KO ESCs. Bar plot showing the mean fold change for the indicated genes compared to wild type, normalized for the housekeeping gene *TBP*. Results of two biological and two technical replicates are plotted. Error bars represent SEM. No significant differences were found (Dunnett’s test).
2. Schematic overview of the dual SMAD NSC differentiation protocol, see methods for details.
3. qRT-PCR showing downregulation of the pluripotency markers *OCT4*, *NANOG* and *REX1* and upregulation of the NSC marker *PAX6* in wild type and *AMFR* KO NSCs at p5 of NSC differentiation. Bar plot showing the mean fold change for the indicated genes compared to wild type, normalized for the housekeeping gene *TBP*. Results of two biological and two independent technical replicates are plotted. Error bars represent SEM (Dunnett’s test, compared with ESC, ****p* < 0.001).
4. Scatterplot depicting the correlation between read counts (in log2(RPKM+1)) of RNA-seq replicates. Pearson correlation coefficient is reported.
5. Heat map visualizing gene expression (in log2(RPKM+1)) of WT and *AMFR* KO NSCs, for a panel of ESC and NSC specific genes, as done previously[38], see methods for details.
6. Enrichment analysis using Enrichr [21] of up- or downregulated genes in *AMFR* KO NSCs for selected gene ontology sets, showing the seven most enriched terms per set. X-axis shows the -log10(p-value) as calculated by Enrichr
7. Enrichment analysis using GSEA for Wikipathway. The upper panel is the density plot of significant enrichments in Wikipathway using the frequency of fold change per gene in the corresponding pathway, the lower panel is the GSEA plot of the cholesterol biosynthesis pathway.
8. qRT-PCR validation of differentially expressed genes from RNA-seq in wild type, and *AMFR* KO NSCs, at p5 of NSC differentiation. Bar plot showing the mean fold change for the indicated genes compared to wild type, normalized for the housekeeping gene *TBP*. Results of two biological and two independent technical replicates from two experiments are plotted. Error bars represent SEM (Kruskal-Wallis test, ***p* < 0.01; ****p* < 0.001).
9. Box-plots showing the counting of the number of Oil Red O (ORO) positive droplets per cell in NSCs. n≥54 cells per genotype in 2 experimental replicates. Boxes represent the interquartile range (IQR); lines represent the median; whiskers extend to 1.5x the IQR; dots represent outliers. (Kruskal-Wallis test, ** p < 0.01).
10. Quantification of the number of ORO positive lipid droplets in KO NSCs transfected with wild type or mutant AMFR. n≥100 cells per genotype in 2 experimental replicates. Boxes represent the interquartile range (IQR); lines represent the median; whiskers extend to 1.5x the IQR; dots represent outliers. (Dunn’s Multiple Comparison test).
11. qRT-PCR expression analysis for UPR marker genes (*HSPA5, IRE1, ATF4,* spliced *XBP1*, and *EDEM*) in wild type and *AMFR* KO NSCs. Shown is the mean fold change for the indicated genes compared to wild type, normalized for the housekeeping gene *TBP*. Results of two biological and two independent technical replicates are plotted, from two experiments. Error bars represent SEM (Kruskal-Wallis test, **p* < 0.05; ***p* < 0.01).
12. qRT-PCR expression analysis for UPR marker genes (*HSPA5, ATF4,* spliced *XBP1*, and *EDEM*) in wild type and *AMFR* KO NSCs upon treatment with DMSO or tunicamycin (at concentration of 2000 ng/µl or 4000 ng/µl). Shown is the mean fold change for the indicated genes compared to DMSO treated wild type, normalized for the housekeeping gene *TBP*. Results of two biological and two independent technical replicates are plotted, from two experiments. Error bars represent SEM (Kruskal-Wallis test, **p* < 0.05; ***p* < 0.01).

**
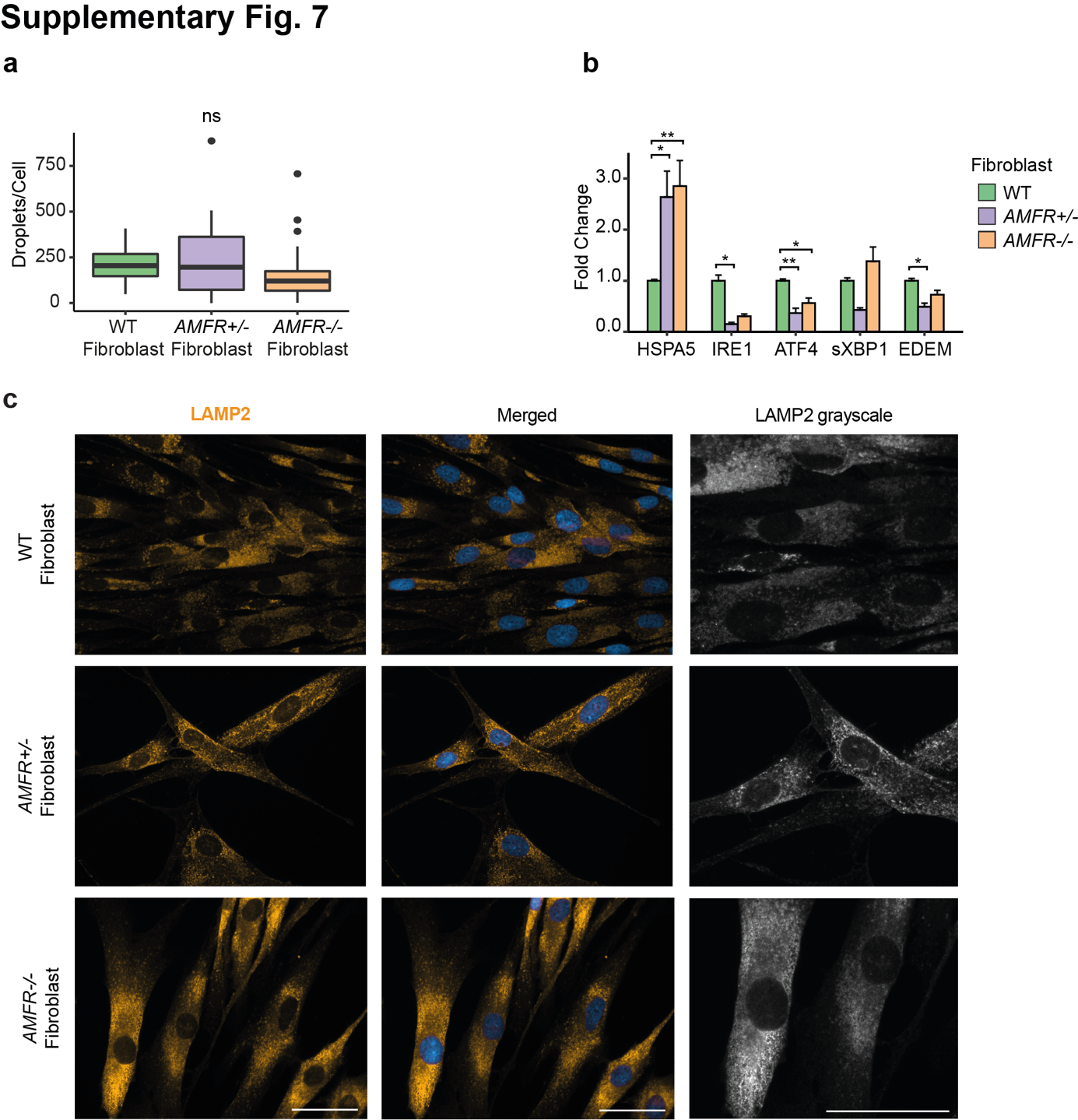
**

## **Supplementary Fig. 7:** Additional analysis of patient-derived fibroblasts.

1. Box-plots showing the counting of the number of Oil Red O (ORO) positive droplets per cell for wild type control fibroblasts (n=1), heterozygous *AMFR* carrier fibroblasts (n=2, from unaffected mothers of family 1 and 8) or homozygous *AMFR* patient-derived fibroblasts (n=3, from individuals 1, 2, and 20), n≥47 cells per sample in 2 experimental replicates. Boxes represent the interquartile range (IQR); lines represent the median; whiskers extend to 1.5x the IQR; dots represent outliers (Dunn’s Multiple Comparison test, ns = not significant).
2. qRT-PCR for ER stress and UPR markers (HSPA5, IRE1, ATF4, sXBP1 and EDEM) in wild type (WT, green) (n=1), heterozygous *AMFR* carrier (from unaffected mothers of family 1 and 8; Het, purple) (n=2) and homozygous *AMFR* patient-derived (from individuals 1, 2, and 20; Hom, orange) (n=3) fibroblasts. Each biological replicate measured in two technical replicates from two independent experiments. Bar plot shows the mean fold change for the indicated genes compared to wild type, normalized for the housekeeping gene TBP. Error bars represent SEM (Dunn’s Multiple Comparison test, **p* < 0.05; ** p<0.01).
3. Representative images of LAMP2 immunostaining performed on the same set of patient-derived fibroblasts and controls. LAMP2 is stained in orange (Cy3) and DAPI in blue. 50 µm scale bar. The analysis did not reveal subcellular differences in staining pattern between patient-derived fibroblasts and controls, which argues that the numerous additional large vesicles seen in EM are not lysosomes.

**
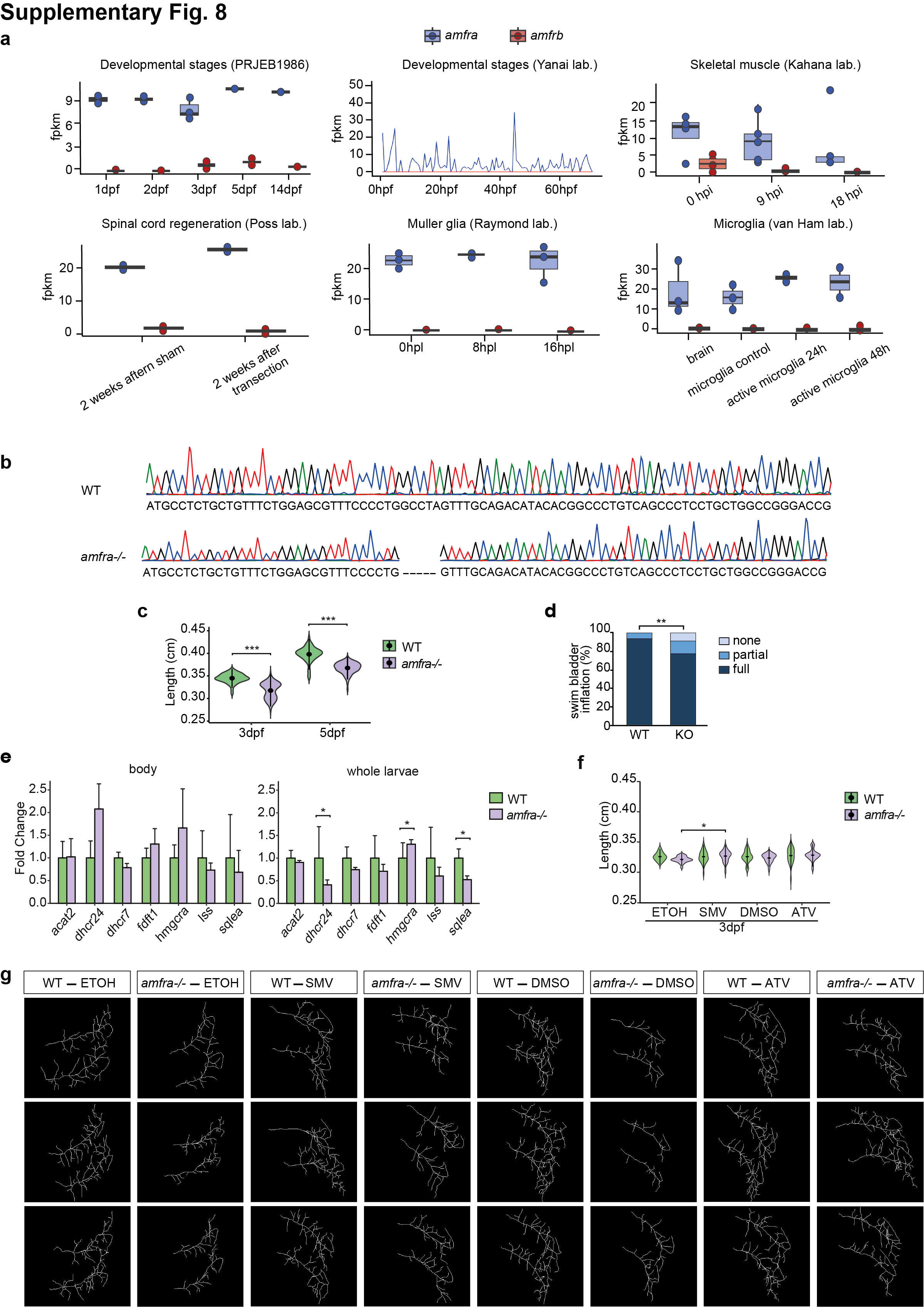
**

## **Supplementary Fig. 8:** Generation and characterization of the *amfra-/-* zebrafish model.

1. Plots showing *amfra* and *amfrb* RNA expression (in FPKM) from previously published zebrafish studies at different developmental stages and time points, publicly available at <http://www.zfregeneration.org/>. Whereas *amfra* is widely expressed at high levels, *amfrb* expression is low to absent at all developmental stages and tissues assessed.
2. Sequencing traces of WT and *amfra-/-* larvae, confirming a homozygous 5 bp deletion in the generated *amfra-/-* strain.
3. Violin plot showing the length at both 3 dpf and 5 dpf of WT and *amfra-/-* larvae (n=40 larvae per genotype, from 2 experimental replicates). WT, green; *amfra-/-,* purple. Black circle, median; black line, SD (Kruskal-Wallis test, ****p* < 0.001).
4. Quantification of the percentage of WT and *amfra-/-* larvae at 5 dpf that have a fully or partially inflated swim bladder or an absent swim bladder (none), n=40 larvae per genotype. At 5 dpf, a small (but statistically significant) fraction of *amfra-/-* larvae have incomplete or absent swim bladder development. Chi-Square test, ***p* < 0.01.
5. qRT-PCR expression analysis for selected cholesterol metabolism genes in bodies or whole larvae of 5 dpf control and *amfra-/-* larvae (for bodies: n=10 bodies per sample, 4 biological replicates for WT larvae and 5 biological replicates for *amfra-/-* and two technical replicates per sample from 2 experiments; for whole larvae: n=15 larvae per sample and 3 biological replicates for each genotype, and two technical replicates per sample from 2 experiments). Bar plot showing the mean fold change for the indicated genes compared to WT larvae, normalized for the housekeeping gene *eef1a1*. WT, green; *amfra-/-* purple. Error bars represent SD (Kruskal-Wallis test, **p* < 0.05).
6. Violin plot showing the length of wild type (WT) and *amfra-/-* larvae at 3 dpf. Larvae are either treated starting from 8 hpf onwards with simvastatin (SMV) or atorvastatin (ATV), or with their respective vehicle controls ethanol or DMSO. n>20 per genotype and treatment group. Black circle, median; black line, SD (Dunn’s Multiple Comparison test, ***p* < 0.01; ****p* < 0.001).
7. Representative images of the ventral motor neurons, detected by acetylated tubulin staining, used for quantification of WT and *amfra-/-* 2 dpf embryos, as shown in main **Figure 4K**. Images show the skeletonized axon output by the SNT plugin in Fiji, representing the branching of axons in the different genotypes and treatment groups with vehicle ETOH or DMSO controls or statin treatment (SMV or ATV).

**
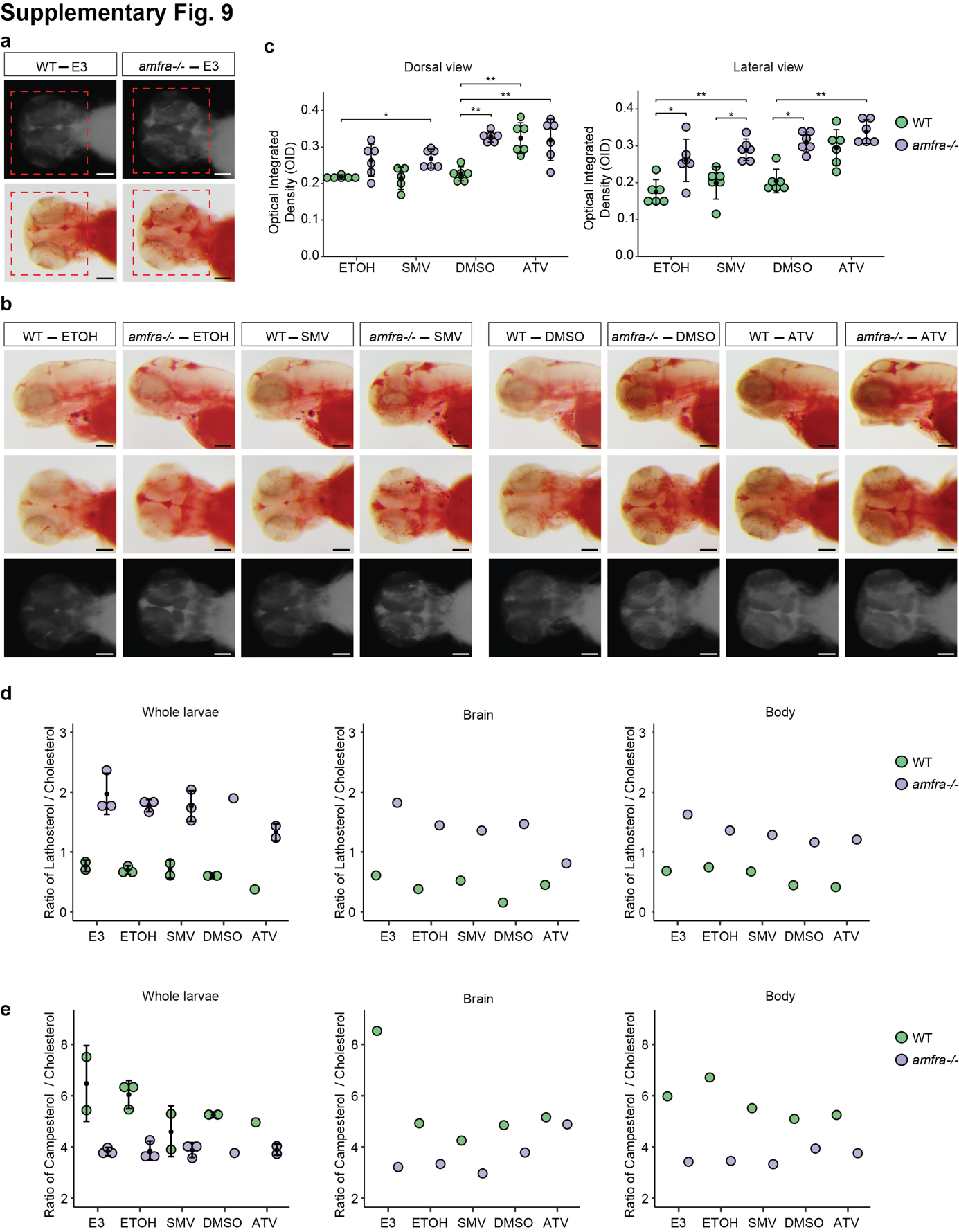
**

## **Supplementary Fig. 9:** Oil Red O staining in zebrafish larvae.

1. Representative images of ORO staining of 3 dpf wild type (WT) and *amfra-/-* larvae, acquired from dorsal view, with the red dotted line indicating the region of interest (ROI) used for quantifications of dorsal view images. Also shown are the color deconvoluted images used for quantification of the ORO stain signal (black and white). Scale bars = 100 µm.
2. Lateral and dorsal view of ORO staining for WT and *amfra-/-* larvae and all treatment groups (vehicle: ETOH or DMSO; and simvastatin (SMV) or atorvastatin (ATV)). Also shown are the color deconvoluted images used for quantification of the ORO staining signal (black and white). Scale bars = 100 µm.
3. Quantification of ORO staining intensity in the heads, from both dorsal (left graph) and lateral view (right graph), of WT and *amfra-/-* larvae at 3 dpf, treated from 8 hpf with simvastatin (SMV) or atorvastatin (ATV) or their respective vehicle controls ethanol (ETOH) or DMSO. Circles show individual values for each larva, n=6 larvae per genotype (WT, green; *amfra-/-*, purple) and treatment, error bars represent SD (Dunn’s Multiple Comparison test, **p* < 0.05; ***p* < 0.01).
4. Graphs showing the ratio between lathosterol and cholesterol concentration, detected in whole larvae (left graph), brain (middle graph) and bodies (right graph) from WT and *amfra-/-* larvae at 5 dpf. Larvae were grown in E3 medium or treated from 8 hpf onwards with simvastatin (SMV) or atorvastatin (ATV) or their respective vehicle controls ethanol (ETOH) or DMSO. Circles show individual values (WT, green; *amfra-/-*, purple), error bars represent SD. Each individual value reflects the measurement of a pool of 7-15 larvae (see **Supplementary Table 5** for the exact numbers per sample) corrected for the number of larvae per pool. Most samples of whole larvae were measured in independent biological replicates, whereas dissected brain and body samples were measured as single pools.
5. Graphs showing the ratio between campesterol and cholesterol concentration, detected in whole larvae (left graph), brain (middle graph) and bodies (right graph) from WT and *amfra-/-* larvae at 5 dpf. Larvae were grown in E3 medium or treated from 8 hpf onwards with simvastatin (SMV) or atorvastatin (ATV) or their respective vehicle controls ethanol (ETOH) or DMSO. Circles show individual values (WT, green; *amfra-/-*, purple), error bars represent SD. Each individual value reflects the measurement of a pool of 7-15 larvae (see **Supplementary Table 5** for the exact numbers per sample) corrected for the number of larvae per pool. Most samples of whole larvae were measured in independent biological replicates, whereas dissected brain and body samples were measured as single pools.

# Overview of separately supplied files:

# Supplementary Table 1-6 and Supplementary Movie 1-4

**Supplementary Table 1**: Extended clinical table, describing all the collected medical information from the 20 individuals with bi-allelic, loss-of-function *AMFR* variants (separate Excel file)

**Supplementary Table 2**: Minor allele frequencies and *in silico* predictions for the encountered *AMFR* variants (separate Excel file)

**Supplementary Table 3**: RNA-seq analysis of wild type and AMFR KO NSCs (separate Excel file)

**Supplementary Table 4**: Functional enrichment analysis of differentially expressed genes (separate Excel file)

**Supplementary Table 5**: Sterol measurements in zebrafish larvae (separate Excel file)

**Supplementary Table 6**: Oligonucleotide sequences used in this study (separate Excel file)

**Supplementary Movie 1**: Walking pattern of individual 3, 4 and 5 from Family 2, showing their spastic gait (separate Movie file)

**Supplementary Movie 2**: The touch-evoked escape response in wild type and *amfra-/-* larvae at 3 dpf (separate Movie file)

**Supplementary Movie 3**: 3D animation showing the axon branching of motor neurons in 2 dpf wild type and *amfra-/-* zebrafish larvae, as obtained from the SNT plugin in Fiji (separate Movie file)

**Supplementary Movie 4**: 3D animation showing the axon branching of motor neurons in 2 dpf wild type and ATV treated *amfra-/-* zebrafish larvae, as obtained from the SNT plugin in Fiji (separate Movie file)

# Supplementary References:

1 The PyMOL Molecular Graphics System, Version 2.0 Schrödinger, LLC.:

2 AlMuhaizea M, AlMass R, AlHargan A, AlBader A, Medico Salsench E, Howaidi J, Ihinger J, Karachunski P, Begtrup A, Segura Castell Met al (2020) Truncating mutations in YIF1B cause a progressive encephalopathy with various degrees of mixed movement disorder, microcephaly, and epilepsy. Acta Neuropathol 139: 791-794

3 Arshadi C, Gunther U, Eddison M, Harrington KIS, Ferreira TA (2021) SNT: a unifying toolbox for quantification of neuronal anatomy. Nat Methods 18: 374-377

4 Barakat TS, Ghazvini M, de Hoon B, Li T, Eussen B, Douben H, van der Linden R, van der Stap N, Boter M, Laven JSet al (2015) Stable X chromosome reactivation in female human induced pluripotent stem cells. Stem Cell Reports 4: 199-208

5 Barakat TS, Halbritter F, Zhang M, Rendeiro AF, Perenthaler E, Bock C, Chambers I (2018) Functional Dissection of the Enhancer Repertoire in Human Embryonic Stem Cells. Cell Stem Cell 23: 276-288 e278

6 Bayer SA, Altman J (2008) Atlas of human central nervous system development: the human brain during the early first trimester. CRC Press, New York 5:

7 Bayer SA, Altman J (2006) Atlas of human central nervous system development: the human brain during the late first trimester. CRC Press, New York 4:

8 Ben-Porath I, Thomson MW, Carey VJ, Ge R, Bell GW, Regev A, Weinberg RA (2008) An embryonic stem cell-like gene expression signature in poorly differentiated aggressive human tumors. Nat Genet 40: 499-507

9 Bhattacharya B, Miura T, Brandenberger R, Mejido J, Luo Y, Yang AX, Joshi BH, Ginis I, Thies RS, Amit Met al (2004) Gene expression in human embryonic stem cell lines: unique molecular signature. Blood 103: 2956-2964

10 Campbell PD, Shen K, Sapio MR, Glenn TD, Talbot WS, Marlow FL (2014) Unique function of Kinesin Kif5A in localization of mitochondria in axons. J Neurosci 34: 14717-14732

11 Carmean V, Ribera AB (2010) Genetic Analysis of the Touch Response in Zebrafish (Danio rerio). Int J Comp Psychol 23: 91

12 Caulfield M, Davies J, Dennys M, et al (2017) The National Genomics Research and Healthcare Knowledgebase v5, Genomics England.:

13 Chai G, Szenker-Ravi E, Chung C, Li Z, Wang L, Khatoo M, Marshall T, Jiang N, Yang X, McEvoy-Venneri Jet al (2021) A Human Pleiotropic Multiorgan Condition Caused by Deficient Wnt Secretion. N Engl J Med 385: 1292-1301 Doi 10.1056/NEJMoa2033911

14 Consortium GT (2013) The Genotype-Tissue Expression (GTEx) project. Nat Genet 45: 580-585

15 Das R, Liang YH, Mariano J, Li J, Huang T, King A, Tarasov SG, Weissman AM, Ji X, Byrd RA (2013) Allosteric regulation of E2:E3 interactions promote a processive ubiquitination machine. EMBO J 32: 2504-2516 Doi 10.1038/emboj.2013.174

16 Dobin A, Davis CA, Schlesinger F, Drenkow J, Zaleski C, Jha S, Batut P, Chaisson M, Gingeras TR (2013) STAR: ultrafast universal RNA-seq aligner. Bioinformatics 29: 15-21

17 Douben HCW, Nellist M, van Unen L, Elfferich P, Kasteleijn E, Hoogeveen-Westerveld M, Louwen J, van Veghel-Plandsoen M, de Valk W, Saris JJet al (2022) High-yield identification of pathogenic NF1 variants by skin fibroblast transcriptome screening after apparently normal diagnostic DNA testing. Hum Mutat:

18 Jiang LY, Jiang W, Tian N, Xiong YN, Liu J, Wei J, Wu KY, Luo J, Shi XJ, Song BL (2018) Ring finger protein 145 (RNF145) is a ubiquitin ligase for sterol-induced degradation of HMG-CoA reductase. J Biol Chem 293: 4047-4055

19 Jo Y, Lee PC, Sguigna PV, DeBose-Boyd RA (2011) Sterol-induced degradation of HMG CoA reductase depends on interplay of two Insigs and two ubiquitin ligases, gp78 and Trc8. Proc Natl Acad Sci U S A 108: 20503-20508

20 Kuil LE, Lopez Marti A, Carreras Mascaro A, van den Bosch JC, van den Berg P, van der Linde HC, Schoonderwoerd K, Ruijter GJG, van Ham TJ (2019) Hexb enzyme deficiency leads to lysosomal abnormalities in radial glia and microglia in zebrafish brain development. Glia 67: 1705-1718

21 Kuleshov MV, Jones MR, Rouillard AD, Fernandez NF, Duan Q, Wang Z, Koplev S, Jenkins SL, Jagodnik KM, Lachmann Aet al (2016) Enrichr: a comprehensive gene set enrichment analysis web server 2016 update. Nucleic Acids Res 44: W90-97

22 Landini G, Martinelli G, Piccinini F (2021) Colour deconvolution: stain unmixing in histological imaging. Bioinformatics 37: 1485-1487

23 Laskowski RA, Jablonska J, Pravda L, Varekova RS, Thornton JM (2018) PDBsum: Structural summaries of PDB entries. Protein Sci 27: 129-134

24 Laskowski RA, Stephenson JD, Sillitoe I, Orengo CA, Thornton JM (2020) VarSite: Disease variants and protein structure. Protein Sci 29: 111-119

25 Lee JN, Song B, DeBose-Boyd RA, Ye J (2006) Sterol-regulated degradation of Insig-1 mediated by the membrane-bound ubiquitin ligase gp78. J Biol Chem 281: 39308-39315

26 Li H, Durbin R (2009) Fast and accurate short read alignment with Burrows-Wheeler transform. Bioinformatics 25: 1754-1760

27 Li W, Tu D, Li L, Wollert T, Ghirlando R, Brunger AT, Ye Y (2009) Mechanistic insights into active site-associated polyubiquitination by the ubiquitin-conjugating enzyme Ube2g2. Proc Natl Acad Sci U S A 106: 3722-3727

28 Liao Y, Smyth GK, Shi W (2014) featureCounts: an efficient general purpose program for assigning sequence reads to genomic features. Bioinformatics 30: 923-930

29 Liberzon A, Subramanian A, Pinchback R, Thorvaldsdottir H, Tamayo P, Mesirov JP (2011) Molecular signatures database (MSigDB) 3.0. Bioinformatics 27: 1739-1740

30 Liu TF, Tang JJ, Li PS, Shen Y, Li JG, Miao HH, Li BL, Song BL (2012) Ablation of gp78 in liver improves hyperlipidemia and insulin resistance by inhibiting SREBP to decrease lipid biosynthesis. Cell Metab 16: 213-225

31 Liu Y, Soetandyo N, Lee JG, Liu L, Xu Y, Clemons WM, Jr., Ye Y (2014) USP13 antagonizes gp78 to maintain functionality of a chaperone in ER-associated degradation. Elife 3: e01369

32 Longair MH, Baker DA, Armstrong JD (2011) Simple Neurite Tracer: open source software for reconstruction, visualization and analysis of neuronal processes. Bioinformatics 27: 2453-2454

33 McKenna A, Hanna M, Banks E, Sivachenko A, Cibulskis K, Kernytsky A, Garimella K, Altshuler D, Gabriel S, Daly Met al (2010) The Genome Analysis Toolkit: a MapReduce framework for analyzing next-generation DNA sequencing data. Genome Res 20: 1297-1303

34 McLaren W, Gil L, Hunt SE, Riat HS, Ritchie GR, Thormann A, Flicek P, Cunningham F (2016) The Ensembl Variant Effect Predictor. Genome Biol 17: 122

35 Menzies SA, Volkmar N, van den Boomen DJ, Timms RT, Dickson AS, Nathan JA, Lehner PJ (2018) The sterol-responsive RNF145 E3 ubiquitin ligase mediates the degradation of HMG-CoA reductase together with gp78 and Hrd1. Elife 7:

36 Mirdita M, Schutze K, Moriwaki Y, Heo L, Ovchinnikov S, Steinegger M (2022) ColabFold: making protein folding accessible to all. Nat Methods 19: 679-682 Doi 10.1038/s41592-022-01488-1

37 Muller JM, Deinhardt K, Rosewell I, Warren G, Shima DT (2007) Targeted deletion of p97 (VCP/CDC48) in mouse results in early embryonic lethality. Biochem Biophys Res Commun 354: 459-465

38 Perenthaler E, Nikoncuk A, Yousefi S, Berdowski WM, Alsagob M, Capo I, van der Linde HC, van den Berg P, Jacobs EH, Putar Det al (2020) Loss of UGP2 in brain leads to a severe epileptic encephalopathy, emphasizing that bi-allelic isoform-specific start-loss mutations of essential genes can cause genetic diseases. Acta Neuropathol 139: 415-442

39 Quinlan AR, Hall IM (2010) BEDTools: a flexible suite of utilities for comparing genomic features. Bioinformatics 26: 841-842

40 Raczy C, Petrovski R, Saunders CT, Chorny I, Kruglyak S, Margulies EH, Chuang HY, Kallberg M, Kumar SA, Liao Aet al (2013) Isaac: ultra-fast whole-genome secondary analysis on Illumina sequencing platforms. Bioinformatics 29: 2041-2043 Doi 10.1093/bioinformatics/btt314

41 Retterer K, Juusola J, Cho MT, Vitazka P, Millan F, Gibellini F, Vertino-Bell A, Smaoui N, Neidich J, Monaghan KGet al (2016) Clinical application of whole-exome sequencing across clinical indications. Genet Med 18: 696-704

42 Robinson MD, McCarthy DJ, Smyth GK (2010) edgeR: a Bioconductor package for differential expression analysis of digital gene expression data. Bioinformatics 26: 139-140

43 Ruifrok AC, Johnston DA (2001) Quantification of histochemical staining by color deconvolution. Anal Quant Cytol Histol 23: 291-299

44 Sanderson LE, Lanko K, Alsagob M, Almass R, Al-Ahmadi N, Najafi M, Al-Muhaizea MA, Alzaidan H, AlDhalaan H, Perenthaler Eet al (2021) Bi-allelic variants in HOPS complex subunit VPS41 cause cerebellar ataxia and abnormal membrane trafficking. Brain 144: 769-780

45 Schindelin J, Arganda-Carreras I, Frise E, Kaynig V, Longair M, Pietzsch T, Preibisch S, Rueden C, Saalfeld S, Schmid Bet al (2012) Fiji: an open-source platform for biological-image analysis. Nat Methods 9: 676-682

46 Snodgrass RG, Zezina E, Namgaladze D, Gupta S, Angioni C, Geisslinger G, Lutjohann D, Brune B (2018) A Novel Function for 15-Lipoxygenases in Cholesterol Homeostasis and CCL17 Production in Human Macrophages. Front Immunol 9: 1906 Doi 10.3389/fimmu.2018.01906

47 Song BL, Sever N, DeBose-Boyd RA (2005) Gp78, a membrane-anchored ubiquitin ligase, associates with Insig-1 and couples sterol-regulated ubiquitination to degradation of HMG CoA reductase. Mol Cell 19: 829-840

48 Sosic-Jurjevic B, Lutjohann D, Renko K, Filipovic B, Radulovic N, Ajdzanovic V, Trifunovic S, Nestorovic N, Zivanovic J, Manojlovic Stojanoski Met al (2019) The isoflavones genistein and daidzein increase hepatic concentration of thyroid hormones and affect cholesterol metabolism in middle-aged male rats. J Steroid Biochem Mol Biol 190: 1-10 Doi 10.1016/j.jsbmb.2019.03.009

49 Subramanian A, Tamayo P, Mootha VK, Mukherjee S, Ebert BL, Gillette MA, Paulovich A, Pomeroy SL, Golub TR, Lander ESet al (2005) Gene set enrichment analysis: a knowledge-based approach for interpreting genome-wide expression profiles. Proc Natl Acad Sci U S A 102: 15545-15550

50 Trujillano D, Bertoli-Avella AM, Kumar Kandaswamy K, Weiss ME, Koster J, Marais A, Paknia O, Schroder R, Garcia-Aznar JM, Werber Met al (2017) Clinical exome sequencing: results from 2819 samples reflecting 1000 families. Eur J Hum Genet 25: 176-182

51 Tsai YC, Leichner GS, Pearce MM, Wilson GL, Wojcikiewicz RJ, Roitelman J, Weissman AM (2012) Differential regulation of HMG-CoA reductase and Insig-1 by enzymes of the ubiquitin-proteasome system. Mol Biol Cell 23: 4484-4494

52 UniProt C (2021) UniProt: the universal protein knowledgebase in 2021. Nucleic Acids Res 49: D480-D489

53 Varadi M, Anyango S, Deshpande M, Nair S, Natassia C, Yordanova G, Yuan D, Stroe O, Wood G, Laydon Aet al (2022) AlphaFold Protein Structure Database: massively expanding the structural coverage of protein-sequence space with high-accuracy models. Nucleic Acids Res 50: D439-D444

54 Walter W, Sanchez-Cabo F, Ricote M (2015) GOplot: an R package for visually combining expression data with functional analysis. Bioinformatics 31: 2912-2914

55 Waqas A, Nayab A, Shaheen S, Abbas S, Latif M, Rafeeq MM, Al-Dhuayan IS, Alqosaibi AI, Alnamshan MM, Sain ZMet al (2022) Case Report: Biallelic Variant in the tRNA Methyltransferase Domain of the AlkB Homolog 8 Causes Syndromic Intellectual Disability. Front Genet 13: 878274

56 Yagishita N, Ohneda K, Amano T, Yamasaki S, Sugiura A, Tsuchimochi K, Shin H, Kawahara K, Ohneda O, Ohta Tet al (2005) Essential role of synoviolin in embryogenesis. J Biol Chem 280: 7909-7916

57 Yu G, Wang LG, Han Y, He QY (2012) clusterProfiler: an R package for comparing biological themes among gene clusters. OMICS 16: 284-287

58 Zhang T, Kho DH, Wang Y, Harazono Y, Nakajima K, Xie Y, Raz A (2015) Gp78, an E3 ubiquitin ligase acts as a gatekeeper suppressing nonalcoholic steatohepatitis (NASH) and liver cancer. PLoS One 10: e0118448

59 Zhou ZS, Li MX, Liu J, Jiao H, Xia JM, Shi XJ, Zhao H, Chu L, Liu J, Qi Wet al (2020) Competitive oxidation and ubiquitylation on the evolutionarily conserved cysteine confer tissue-specific stabilization of Insig-2. Nat Commun 11: 379
